# The Overlooked Dual Phosphorescence of Lappert's Diamino Stannylene Sn[N(SiMe_3_)_2_]_2_

**DOI:** 10.1002/anie.202510044

**Published:** 2025-07-21

**Authors:** Philipp Sikora, Robert Naumann, Lukas Sorge, Christoph Förster, Katja Heinze

**Affiliations:** ^1^ Department of Chemistry Johannes Gutenberg University Mainz Duesbergweg 10–14 55128 Mainz Germany

**Keywords:** Excimer, Phosphorescence, Radicals, Stannylene, Transient absorption spectroscopy

## Abstract

The first stable heavy carbene homologues, the heavy tetrylenes, were reported in 1973 by Lappert and coworkers. These tetrylenes were extensively investigated with respect to ground state reactivity, such as small molecule activation, insertion into σ‐bonds, coordination chemistry, materials chemistry, or catalysis. Their photophysical properties remained essentially unexplored. We report that the bright yellow‐colored diamino stannylene Sn[N(SiMe_3_)_2_]_2_ shows thermally activated dual orange/green phosphorescence with microsecond lifetime in fluid solution at room temperature, which has been overlooked for more than 50 years. These unique electronic and photophysical properties are studied in detail by temperature‐dependent time‐resolved emission and absorption spectroscopy and are corroborated by (time‐dependent) density functional theory (DFT) calculations. The mechanism of photochemical radical formation has been disclosed, involving unprecedented stannylene excimers with second‐order Jahn–Teller distorted structures. The present study provides new insights toward a rational design of tetrel(II) complexes with long‐lived emissive excited states, with Sn[N(SiMe_3_)_2_]_2_ being the prototype.

## Graphical abstract

The diamino stannylene Sn[N(SiMe_3_)_2_]_2_ shows rich photophysics and photochemistry: unprecedented dual green/orange phosphorescence with long excited state lifetimes, excimer formation, and unimolecular bond homolysis. Photolytic Sn─N bond homolysis occurs only in the long‐lived excited monomer but not in the excimer.

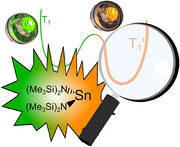

## Introduction

Great progress has been achieved in the research of phosphorescent and photoactive transition metal complexes of the Earth‐abundant elements, mainly 3d elements, and evolved to a potential alternative to complexes of the precious transition metals, e.g., ruthenium, iridium, or platinum.^[^ ^1^, ^2^, ^3^, ^4^, ^5^, ^6^ ^]^ For photochemical applications, the prerequisite is a sufficiently long lifetime of the excited states (ESs), which is often fulfilled for ESs possessing different spin multiplicities than the ground state (GS). Radiative and nonradiative processes become spin‐forbidden, extending the ES lifetime. Efficient intersystem crossing (ISC) after initial population of the Franck–Condon state provides access to ESs of different spin multiplicity. The ISC rate becomes large for strong spin‐orbit coupling (SOC), often induced by a so‐called heavy‐atom effect, by direct SOC described by the qualitative El‐Sayed rules,^[^ ^7^, ^8^ ^]^ generalized by Marian,^[^ ^9^ ^]^ or by spin‐vibronic coupling.^[^ ^9^, ^10^ ^]^ According to the energy‐gap law, the ESs should be rather nested, i.e., undistorted, for a long ES lifetime and not too low in energy.^[^ ^11^ ^]^ While suitable design concepts for strongly luminescent transition metal complexes have been developed, such as increasing the ligand field strength, rigidification for d^10^ complexes and prevention of self‐quenching,^[^ ^2^, ^3^, ^5^, ^12^, ^13^, ^14^, ^15^, ^16^, ^17^, ^18^, ^19^ ^]^ analogous rules and structure–property relationships are missing for phosphorescent heavy main group compounds.

**Scheme 1:**
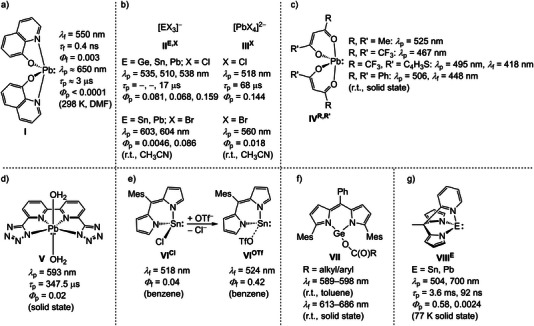
Photoluminescent tetrel(II) complexes with selected photophysical data: phosphorescent a) 8‐quinolinolato plumbylene,^[^ ^34^ ^]^ b) halido tetranides,^[^ ^35^, ^36^ ^]^ c) β‐diketonato plumbylenes,^[^ ^37^ ^]^ d) diaqua tetrazolato lead(II) complex,^[^ ^38^ ^]^ fluorescent e) dipyrrinato chlorido/triflato stannylenes,^[^ ^39^ ^]^ f) dipyrrinato carboxylato germylenes,^[^ ^40^ ^]^ and phosphorescent g) stannylene and plumbylene with tridentate dipyrrolato‐pyridine ligand.^[^ ^41^ ^]^

Heavy main group complexes, especially with groups 14 and 15 elements showing phosphorescence and/or thermally activated delayed fluorescence (TADF), are rather rare.^[^ ^20^, ^21^, ^22^, ^23^ ^]^ Phosphorescence has been reported for bismuth(III) complexes with 6s^2^ electron configuration,^[^ ^24^, ^25^, ^26^, ^27^, ^28^, ^29^, ^30^, ^31^, ^32^, ^33^ ^]^ while photoluminescent isoelectronic group 14 complexes with the tetrel atom in the oxidation state +II are very scarce. The ESs are typically short‐lived with only weak emission, often limited to frozen solution or to the solid state (**I**−**VIII^E^**, Scheme 1).^[^ ^34^, ^35^, ^36^, ^37^, ^38^, ^39^, ^40^, ^41^ ^]^ Our group investigated the ES landscape of pyrrolato tetrel(II) complexes E(bpep) (**VIII^E^**, E = Sn, Pb, H_2_bpep = 2‐[1,1‐bis(1*H*‐pyrrol‐2‐yl)ethyl]pyridine) with the tin complex surpassing the lead congener in terms of photophysical performance. **VIII^Sn^** shows non‐Kasha phosphorescence from a triplet intraligand CT (^3^ILCT) state.^[^ ^41^ ^]^ Yet, this ^3^ILCT state deactivates very efficiently at room temperature in fluid solution and in the solid state via a lower‐lying, strongly distorted ligand‐to‐metal CT (^3^LMCT) state leading to Sn─N^py^ bond cleavage.^[^ ^41^ ^]^

A deeper understanding of the structural, electronic, and reactivity properties of these tetrel +II compounds (heavy tetrylenes, i.e., heavy carbene homologues^[^ ^42^, ^43^, ^44^, ^45^, ^46^, ^47^ ^]^) might give a rationale for developing photoactive tetrel +II compounds. Therefore, we set out to explore the undeveloped photophysics and photochemistry of literature‐known tetrylenes based on their well‐explored GS properties.

**Figure 1:**
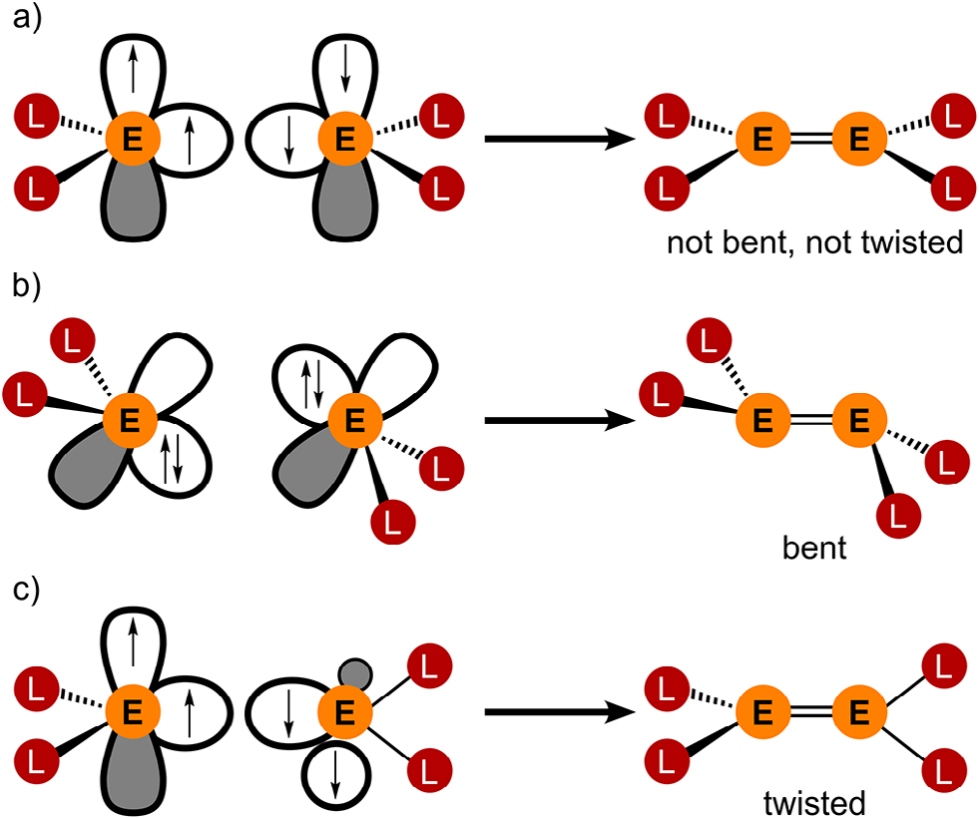
Geometries of ditetrenes from a) two triplet tetrylenes, b) two singlet tetrylenes, and c) two triplet tetrylenes with bulky ligands L (adapted from Ref. [57]).

Typically, the GS of heavy tetrylenes is a singlet state,^[^ ^42^, ^43^, ^44^, ^45^, ^46^, ^47^, ^48^ ^]^ based on the increasing radial and energetic separation of ns/np orbitals from C to Pb.^[^ ^49^ ^]^ The GS wavefunction of the heavier tetrylenes is characterized by a tetrel‐centered fully occupied and a vacant orbital of high s‐ and p‐character, respectively.^[^ ^43^ ^]^ Seminal reports of Lappert and coworkers on stable, isolable heavy tetrylenes started in 1973 with the report of E[CH(SiMe_3_)_2_]_2_ (E = Sn, Pb),^[^ ^50^ ^]^ followed by E[N(SiMe_3_)_2_]_2_ (E = Ge − Pb).^[^ ^51^, ^52^ ^]^ Sn[CH(SiMe_3_)_2_]_2_ forms dimers (distannenes) with a *trans*‐bent structure in the solid state.^[^ ^53^, ^54^ ^]^ This *trans*‐bent or *trans*‐pyramidalized geometry together with a twisted arrangement of the SnR_2_ moieties is a common structural feature of distannenes (Figure 1).^[^ ^55^, ^56^, ^57^ ^]^ In contrast to alkenes with a classical C═C double bond (*σ*‐ and *π*‐bond), the *trans*‐pyramidalization of distannenes^[^ ^43^, ^44^, ^47^, ^55^, ^56^ ^]^ is ascribed to a double Lewis acid–base interaction of two singlet SnR_2_ fragments.^[^ ^54^ ^]^ This binding motif originates in the increasing singlet‐triplet energy difference from CR_2_ to PbR_2_, due to the increasing ns/np‐orbital separation^[^ ^49^ ^]^ with concomitant increase in Pauli repulsion.^[^ ^58^ ^]^ The tendency to dimerize decreases from CR_2_ to PbR_2_.^[^ ^44^, ^48^, ^56^, ^59^, ^60^ ^]^

The change from *D*_2h_ (planar) to *C*_2h_ (*trans*‐bent) symmetry of the R_2_E═ER_2_ core arises from a second‐order Jahn–Teller (SOJT) effect (Figure 1).^[^ ^44^, ^48^, ^61^, ^62^ ^]^ Additionally, the SnR_2_ moieties of sterically congested systems, such as dihypersilyl stannylene Sn[Si(SiMe_3_)_3_]_2_ and systems with small singlet‐triplet gaps are twisted.^[^ ^57^, ^63^, ^64^, ^65^ ^]^ Quantum chemical investigations revealed that the dimerization is driven by significant contributions from strong electrostatic interactions and London dispersion forces.^[^ ^66^, ^67^, ^68^ ^]^ Apart from few examples,^[^ ^57^, ^65^, ^69^, ^70^, ^71^, ^72^ ^]^ the Sn═Sn bonds are weak and commonly the distannenes dissociate in solution.^[^ ^43^, ^44^, ^47^, ^55^, ^56^ ^]^ Clearly, photophysics will depend on the electronic structure of the tetrylenes and their possible dimerization in the GS and ESs, but their photophysics is basically unknown.

While the archetype diamino stannylene Sn[N(SiMe_3_)_2_]_2_ (**Sn**) has been the focus of research for decades now, its photophysics is underexplored to date. Although this bright yellow‐colored, thermally stable^[^ ^73^ ^]^ stannylene shows thermochromism (to dark orange color),^[^ ^52^, ^73^ ^]^ it remains monomeric in solution as determined by cryoscopy^[^ ^52^ ^]^ and NMR spectroscopy.^[^ ^74^ ^]^ In contrast to the dialkyl stannylene Sn[CH(SiMe_3_)_2_]_2_, the diamide **Sn** is also monomeric in the solid state.^[^ ^75^ ^]^ Nevertheless, the molecules are arranged as *trans*‐bent centrosymmetric pairs with a 4.95 Å Sn⋯Sn distance (space group *P*ccn).^[^ ^76^ ^]^ The Lewis‐amphoteric **Sn** acts as ligand in transition metal complexes with subsequent transformations^[^ ^77^, ^78^, ^79^, ^80^ ^]^ and engages in addition and insertion reactions in the GS.^[^ ^81^, ^82^, ^83^, ^84^, ^85^, ^86^, ^87^ ^]^ Further, **Sn** is a valuable precursor to access new stannylenes in salt metathesis and transamination reactions.^[^ ^41^, ^53^, ^63^, ^88^, ^89^, ^90^ ^]^ Thanks to its importance in small molecule activation, insertion into σ‐bonds, coordination chemistry, materials chemistry, or catalysis, **Sn** is meanwhile available in good yields on a large scale.^[^ ^91^ ^]^

**Scheme 2:**
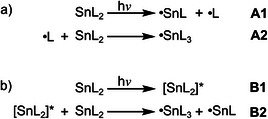
Possible routes for the formation of the tin(III) radical •SnL_3_ as proposed by Lappert and coworkers.^[^ ^93^ ^]^

Upon irradiation with UV light, **Sn** disproportionates, forming the persistent tin(III) radical •Sn[N(SiMe_3_)_2_]_3_.^[^ ^73^, ^92^, ^93^ ^]^ Similarly, Sn[CH(SiMe_3_)_2_]_2_ transforms to •Sn[CH(SiMe_3_)_2_]_3_ by UV photolysis.^[^ ^92^, ^93^, ^94^ ^]^ While the lead congeners could not be generated photochemically,^[^ ^93^ ^]^ persistent/isolable lead(III) radicals can be obtained redox chemically.^[^ ^95^, ^96^, ^97^ ^]^ Numerous examples of •EL_3_ radicals (E = Si─Sn) have been reported.^[^ ^98^, ^99^, ^100^, ^101^ ^]^ Lappert and coworkers proposed two possible pathways for the photochemical generation of •SnL_3_ radicals (L = alkyl, amino) from the respective stannylenes (Scheme 2).^[^ ^93^ ^]^ Mechanism A (Scheme 2a) starts with the photoinduced homolysis of a tin─ligand bond in SnL_2_ (A1) and the free ligand radical •L is trapped by a further SnL_2_ in the GS forming the tin(III) radical •SnL_3_ (A2). Mechanism B (Scheme 2b) is a bimolecular photoreaction between an electronically excited stannylene [SnL_2_]* (B1), tentatively in the triplet state, which reacts with a second SnL_2_ in the GS (B2). A tin(I) radical •SnL, formed in both mechanisms, could not be detected either directly or via trapping.^[^ ^93^, ^94^ ^]^ However, the fate of these elusive species could be elucidated by chemical generation of •Sn[CH(SiMe_3_)_2_] with subsequent formation of a hexastannylprismane Sn_6_[CH(SiMe_3_)_2_]_6_.^[^ ^102^ ^]^

Photolysis of stannylenes with sterically very demanding ligands, such as Sn[NR_2_]_2_ (R = SiEt_3_, GePh_3_), did not furnish the respective tin(III) radicals but allowed the detection of an aminyl radical for R = SiEt_3_, supporting mechanism A (Scheme 2).^[^ ^73^ ^]^ Formation of tin(I) radicals without detection of tin(III) radicals after photolysis of the sterically congested Sn[Ar*^i^* ^Pr4^]_2_ (Ar*^i^* ^Pr4^ = C_6_H_3_‐2,6‐(C_6_H_3_‐2,6‐*^i^* Pr_2_)_2_)^[^ ^103^ ^]^ was reported in addition, supporting mechanism A as well (Scheme 2). On the other hand, mechanism B cannot be ruled out a priori for **Sn** on the basis of existing experimental results.

Importantly, the occurrence of a possible bimolecular photoreaction would be a clear indication that ESs of these prototypical monomeric stannylenes must be long‐lived—at least in the nanosecond regime for a diffusion‐controlled bimolecular reaction. For this reason, we chose the thermally stable diamino stannylene **Sn** for deep photophysical investigations. New insights into the photophysical/chemical properties are presented in this detailed experimental and computational study, revealing a so far overlooked strong dual photoluminescence, excimer formation, and ES SOJT effects. The mechanism of the photochemical •Sn[N(SiMe_3_)_2_]_3_ radical formation is further underpinned. The present work includes UV–vis absorption, time‐resolved emission, and ns/fs‐transient absorption (TA) spectroscopy at variable temperatures and excitation energies, supported by detailed (time‐dependent) density functional theory ((TD)DFT) calculations.

## Results and Discussion

### Photophysical Properties

**Figure 2:**
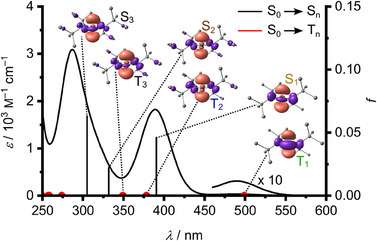
Absorption spectrum at 293 K of **Sn** in *n*‐pentane (*c* = 0.61 mM) at 293 K with TDDFT calculated oscillator strengths for S_0_→S*_n_* transitions, position of S_0_→T*_n_* transitions marked by red points and difference electron densities of selected transitions (isosurface value 0.005 a.u., purple = electron loss, orange = electron gain. CPCM(hexane)‐RIJCOSX‐B3LYP‐D3BJ‐SARC/J‐ZORA/def2‐TZVPP/SARC‐ZORA‐TZVPP(Sn)).

The UV–vis absorption spectrum of **Sn** in *n*‐pentane at room temperature shows two intense bands at 287 and 389 nm (*ε* = 3080 and 1830 M^−1^ cm^−1^) and a much weaker band at 490 nm (*ε* = 31 M^−1^ cm^−1^; Figure 2 and Table 1), in accordance to an earlier report.^[^ ^52^ ^]^ All absorption bands sharpen and slightly shift hypsochromically upon cooling to 77 K (Table 1 and Figure S3). The band at 389 nm was assigned to a metal‐centered (MC) transition from the metal lone‐pair to the metal p‐type orbital by Lappert and coworkers.^[^ ^73^ ^]^ However, our TDDFT calculations on the geometry relaxed structure of **Sn** give a more differentiated picture. Both strong bands at 287 and 389 nm belong to spin‐allowed electronic transitions (S_0_→S_n_), characterized by mainly LMCT(N→Sn)/MC contributions as determined from charge transfer number analysis (Figures 2 and S4; Table 1). The weak band at 490 nm is assigned to a spin‐forbidden S_0_→T_1_ transition of mixed MC/LMCT character. The presence of a spin‐forbidden S_0_→T_1_ absorption band with appreciable intensity suggests that SOC must be very effective in **Sn**. SOC is induced by the heavy‐atom effect with a significant metal character in the T_1_ state (Figure 2, SOC constant ζ(Sn) = 1855 cm^−1^).^[^ ^104^ ^]^ For comparison, the SOC constants for platinum and osmium, showing appreciable S_0_→T_1_ absorption bands in their prototypical complexes amount to 4481 and 3381 cm^−1^, respectively.^[^ ^104^, ^105^, ^106^, ^107^ ^]^

**Table 1:** Optical properties of **Sn**.

*T* (K)	*λ*_abs_ (nm)	*ε* (M^−1^ cm^−1^)	*λ*_em_ (nm)	*τ*_p_	*Φ*
293a)	287, 389, 490	3080, 1830, 31	650	1.2 µsb), 16 nsc)	0.018b)
77d)	283, 310 (sh), 381, 480	n.d.	555	25 µs	n.d.
293e)	n.d.	n.d.	605	8.6 µs	0.2f)
77e)	n.d.	n.d.	550	21 µs	0.8f)

n.d.: not detectable.

^a)^ In *n*‐pentane.

^b)^ *c* = 0.32 mM (*λ*_exc_ = 375/390 nm).

^c)^ *c* = 25 mM (*λ*_exc_ = 450 nm).

^d)^In 3‐methylpentane.

^e)^In the solid state.

^f)^ *λ*_exc_ = 480 nm.

Assuming an idealized *C*_2v_ geometry of the N─Sn─N core, the S_0_→S_1_/T_1_, S_0_→S_2_/T_2_ and S_0_→S_3_/T_3_ transitions are mainly of HOMO(a_1_)/HOMO−1(b_2_)/HOMO−2(a_2_)→LUMO(b_2_) character, respectively (Table S1). Group theoretical considerations identify S_1_/T_1_, S_2_/T_2_, and S_3_/T_3_ as states with ^1/3^B_2_, ^1/3^A_1_, and ^1/3^B_1_ term symbols, respectively. As SOC is expected between states, which are connected via rotations,^[^ ^108^ ^]^ namely S_0_(^1^A_1_)↔T_1_(^3^B_2_)/T_3_(^3^B_1_), S_1_(^1^B_2_)↔T_2_(^3^A_1_)/T_3_(^3^B_1_), S_2_(^1^A_1_)↔T_1_(^3^B_2_)/T_3_(^3^B_1_). These considerations together with the experimental observation of the S_0_→T_1_ transition are supported by SOC‐TDDFT calculations with non‐negligible SOC constants and with significant oscillator strengths for the S_0_↔T_1_ transitions at the Franck–Condon geometry (Tables S2 and S3).

**Figure 3:**
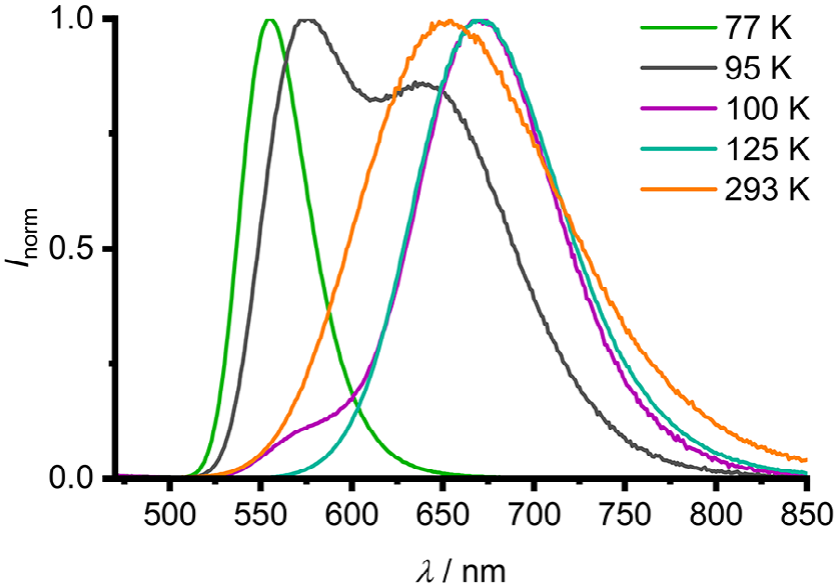
Normalized temperature‐dependent photoluminescence spectra of **Sn** in 3‐methylpentane between 77 K (green) and 293 K (orange) with *λ*_exc_ = 390 nm.

Spin‐allowed S_0_→S_1_ excitation of **Sn** at *λ*_exc_ = 390 nm furnishes green strong and long‐lived T_1_→S_0_ phosphorescence peaking at 555 nm with *τ* = 25 µs lifetime in frozen 3‐methylpentane solution at 77 K (Figure 3, green spectrum). The S_0_→T_1_ (480 nm)/T_1_→S_0_ (555 nm) Stokes shift amounts to 2820 cm^−1^ (Figures 3 and S5; Table 1).

At 95 K, the green emission band shifts to 560 nm and a second, orange phosphorescence band (T_1_’→S_0_’) appears peaking at 640 nm (Figure 3). The exact nature of the involved singlet and triplet states will be discussed below. Upon raising the temperature 95 K→100 K→125 K, where the sample solution is fluid for the latter, the green emission band vanishes and the orange emission band bathochromically shifts to 670 nm (Figure 3). This behavior is fully reversible. At 293 K in fluid solution, **Sn** is still emissive with a broad band peaking at 650 nm. The excitation spectrum follows the absorption spectrum confirming that the emission stems from **Sn** (Figure S6). Band shape analysis with Voigt functions delivers two underlying bands with maxima at 643 and 679 nm, resembling the (rigidochromically shifted) T_1_→S_0_ and T_1_’→S_0_’ bands (Figures 3 and S6−S9). This suggests that the emissive T_1_ and T_1_’ states thermally equilibrate via (back) internal conversion (IC/bIC) at 293 K.

With increasing concentration from 0.32 to 25 mM at 293 K, the photoluminescence lifetime drastically drops from 1.2 µs to 16 ns (Table 1). This is a clear indication of dynamic self‐quenching, tentatively assigned to excimer formation, similar to the aggregation of square‐planar platinum(II) complexes.^[^ ^16^, ^17^, ^18^ ^]^ Stern–Volmer analysis from photoluminescence lifetime measurements yields a quenching rate constant of *k*_q_ = 2.3 10^9^ M^−1^ s^−1^ close to the diffusion limit. Additional static quenching (pre‐association of ground state molecules),^[^ ^109^ ^]^ as observed for platinum(II) and gold(III) complexes,^[^ ^16^, ^17^, ^18^, ^19^ ^]^ can be ruled out as steady‐state emission measurements show a linear concentration dependence as well. This suggests exclusive dynamic quenching in this concentration range (Figures S10 and S11). The absence of static luminescence quenching by dimer formation is in full accordance to earlier reports on the monomeric nature of **Sn** in solution (cryoscopy, NMR spectroscopy).^[^ ^52^, ^74^ ^]^

The natural photoluminescence lifetime in the absence of self‐quenching at the *c*→0 M limit is estimated as *τ*_0_ = 9.5 µs (*k*_0_ = 105 000 s^−1^, *y* axis intercept) at 293 K (Figure S10). The photoluminescence quantum yield determined at low concentration of 0.32 mM amounts to *Φ* = 0.018 at 293 K (Table 1). The intrinsic quantum yield in the absence of self‐quenching for *c*→0 M is determined as *Φ*_0_ = 0.14. The respective nonradiative rate constant is obtained as *k*_nr,0_ = *k*_0_–*k*_r_ = 90 000 s^−1^ from the radiative rate constant of *k*_r_ = *Φ*/*τ* = 15 000 s^−1^. The radiative rate constant *k*_r_ of **Sn** ranges in between that of phosphorescent chromium(III) complexes (*k*_r_ ≈ 100 s^−1^, Laporte‐forbidden MC transition, weak SOC) and prototypical [Ru(bpy)_3_]^2+^ (*k*_r_ ≈ 110 000 s^−1^, ^3^MLCT emission, strong SOC),^[^ ^110^ ^]^ indicating that SOC operates significantly in **Sn**.

**Figure 4:**
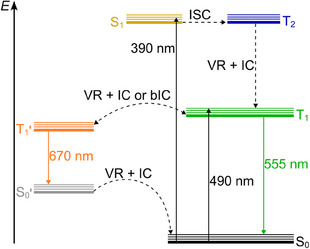
Jablonski diagram of **Sn** with relevant photophysical processes.

The combined data on the temperature‐dependent population of the T_1_/T_1_’ states indicate that T_1_ and the slightly lower‐energy T_1_’ state are connected by a barrier so that at 293 K both states are accessible by IC and bIC. The combined data allow to assemble a Jablonski‐type diagram including relevant photophysical processes (Figure 4). After S_0_→S_1_ excitation, S_1_→T_2_ ISC occurs with high probability according to the similar S_1_/T_2_ energies and the calculated large SOC constant (Table S2). After subsequent T_2_→T_1_ IC and vibrational relaxation (VR), green non‐Kasha T_1_→S_0_ phosphorescence is observed at 77 K. At elevated temperatures, thermally activated T_1_→T_1_’ IC becomes operative with subsequent orange T_1_’→S_0_’ phosphorescence. At 293 K, dual T_1_’→S_0_’/T_1_→S_0_ phosphorescence is observed via thermally activated T_1_’→T_1_ bIC, with T_1_ and T_1_’ states being in thermal equilibrium.

**Figure 5:**
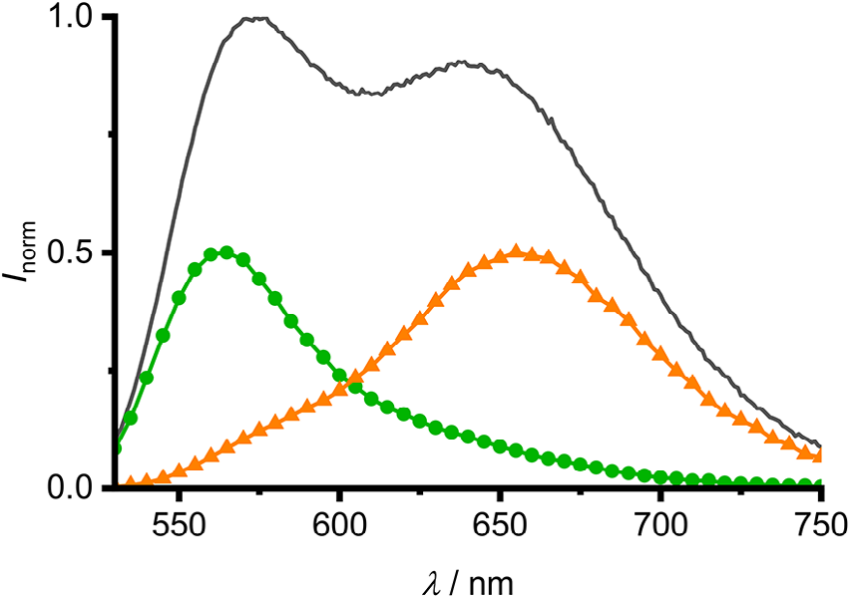
Normalized emission spectrum of **Sn** at 95 K in 3‐methylpentane in grey, normalized time‐resolved emission spectra from time‐averaged spectra between 1–2 µs (green) and 47–60 µs (orange) (*λ*_exc_ = 450 nm, S_0_→T_1_).

To gain deeper insight into the dynamics of the photophysical processes between T_1_ and T_1_’, time‐resolved emission spectroscopy (TRES) was performed at 95 K, where both emission bands are similar in intensity with well‐resolved band maxima (Figures 3 and 5). The TRES scan with direct S_0_→T_1_ excitation (*λ*_exc_ = 450 nm) reveals that the green T_1_ emission appears solely within the first 5 µs and further evolves to the orange T_1_’ emission (Figures S12 and S13).

Global analysis yields four time constants *τ*_1_−*τ*_4_. Several conformers might be local minima on the 3N−6 dimensional hypersurface, slightly differing from the minimum geometry of the S_0_ state. Such slightly distorted geometries might be present in the frozen solution. According to the evolution‐associated spectra, we tentatively assign *τ*_1_ = 1 and *τ*_2_ = 5 µs to decays of states with similar T_1_ character, but with small geometry differences (Figure S13). *τ*_3_ = 13 µs might originate from an intermediate conformer during the T_1_→T_1_’ conversion and *τ*_4_ = 26 µs is ascribed to the T_1_’ state decay. The steady‐state emission spectrum at 95 K with direct S_0_→T_1_ excitation at the high energy tail (*λ*_exc_ = 450 nm) coincides with that obtained from S_0_→S_1_ excitation (*λ*_exc_ = 390 nm; Figures 3, 5, and S3). These data indicate that the T_1_/T_1_’ population transfer starts in the T_1_ state (as shown in Figure 4) and is not a result of branching in higher‐energy singlet or triplet states. The steady‐state emission spectrum consists of the time‐averaged spectra within the 1−2 (T_1_ emission) and 47−60 µs (T_1_’ emission) ranges. This clearly demonstrates the initial T_1_ population with subsequent T_1_→T_1_’ IC (Figures 3, 4, 5).

**Figure 6:**
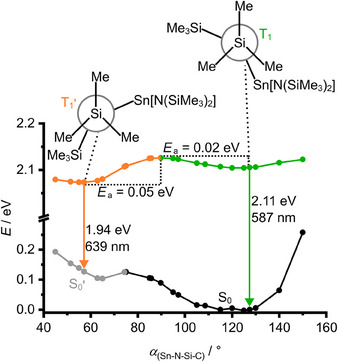
Relaxed potential energy surface scan of **Sn** as projection along the rotation of a trimethylsilyl group, described by the dihedral angle *α*(Sn─N─S─C) with selected Newman projections of the T_1_ state (*α* = 127°) in green and T_1_’ state (*α* = 57°) in orange with single point energies on singlet GS hypersurface at triplet geometries. CPCM(hexane)‐RIJCOSX‐B3LYP‐D3BJ‐SARC/J‐ZORA/def2‐TZVPP/SARC‐ZORA‐TZVPP(Sn).

To gain insight into the distinct nature of the T_1_ and T_1_’ states, relaxed potential energy surface scans on the triplet hypersurface were conducted using DFT. Two local minima were localized. The relaxed geometries differ merely by the orientation of one SiMe_3_ group described by the dihedral angle *α*(Sn─N─Si─C) (Figure 6), while the electronic structure of the two conformers T_1_ and T_1_’ remains unperturbed with nearly identical spin density distributions (Figure S14). The DFT energies for the vertical T_1_→S_0_/T_1_’→S_0_’ transitions (2.11 eV (587 nm)/1.94 eV (639 nm) fit very well to the experimental observed emission energies at 95 K (2.21 eV (560 nm)/1.91 eV (650 nm); Figures 5 and 6). A transition state connects the T_1_ and T_1_’ state minima via a small T_1_→T_1_’ barrier of 0.02 eV as obtained from DFT calculations (Figure S14). The sufficiently small T_1_’→T_1_ barrier of 0.05 eV allows bIC at 293 K (Figure 6). S_0_ is stabilized with respect to S_0_’ by Δ*G* = −5.8 kJ mol^−1^ (−0.06 eV), suggesting a negligible thermal population of the S_0_’ state.

**Figure 7:**
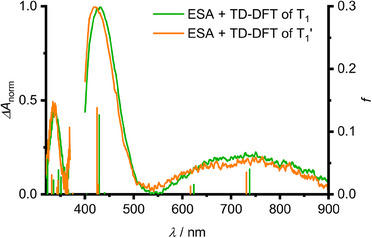
Normalized, time‐averaged ns‐TA spectra of **Sn** at 95 K in 3‐methylpentane of T_1_ (0.3–2 µs) and T_1_’ (8–20 µs) after S_0_→S_1_ excitation at *λ*_pump_ = 390 nm with TDDFT calculated oscillator strengths, shifted by 0.5 eV to higher energies, at the relaxed triplet geometries in green and orange, respectively.

To further substantiate the proposed assignments and dynamics, we employed nanosecond and femtosecond transient absorption (ns/fs‐TA) spectroscopy at different temperatures and with different excitation wavelengths in conjunction with TDDFT calculations. The time‐averaged ns‐TA spectrum at 95 K at early times (0.3–2 µs) features one broad ES absorption (ESA) in the visible and two sharper ESAs in the UV spectral region. Within 8–20 µs, the ESA bands shift hypsochromically while retaining the ESA band pattern (Figure 7). Assigning the spectra at early and late times to the T_1_ and T_1_’ states, their electronic nature must be essentially identical, as noted above. TDDFT calculations of geometry relaxed T_1_ and T_1_’ states essentially reproduce the ns‐TA spectra and describe the observed hypsochromic shift of the ESA bands very well. This agreement further supports the assignment of T_1_ and T_1_’ to different conformers (Figure 7). The photophysical dynamics obtained from ns‐TA measurements match to those from TRES (Figures S12, S13, S15, and S16). The time‐averaged ns‐TA spectrum at 293 K is hence composed of the ESAs of the T_1_ and T_1_’ states in full agreement with the thermally activated dual T_1_→S_0_/T_1_’→S_0_’ phosphorescence at 293 K (Figures 3 and S17).

**Figure 8:**
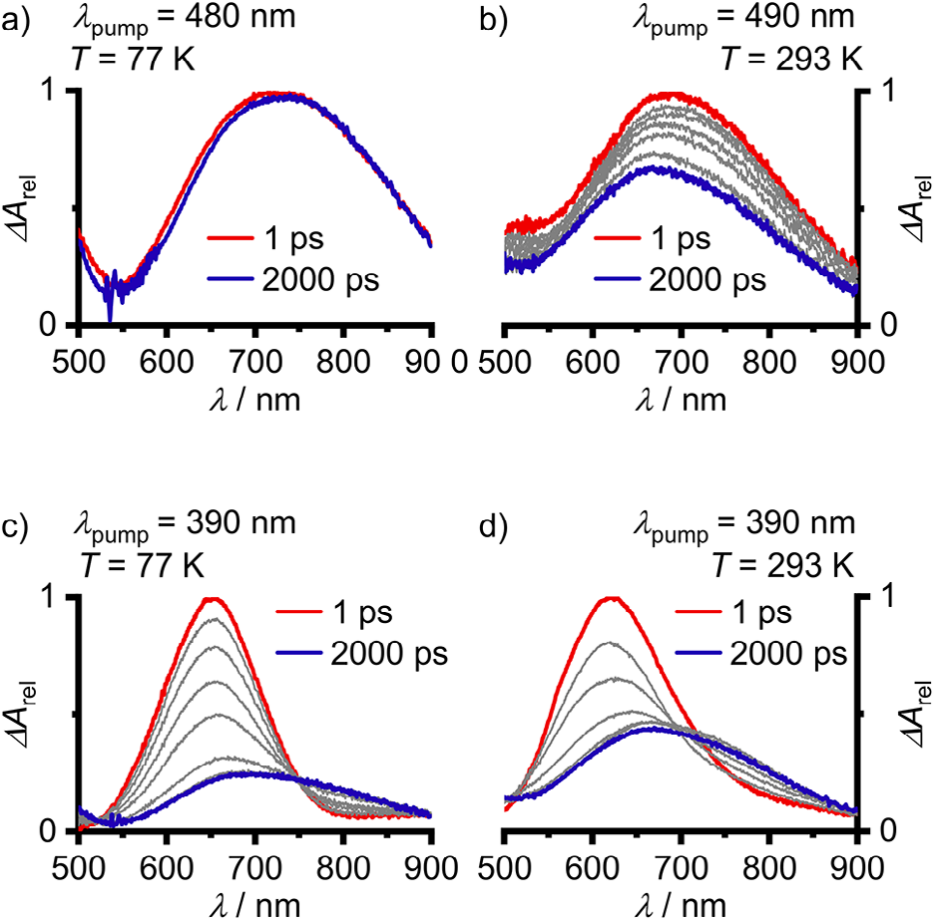
fs‐TA spectra of **Sn** in 3‐methylpentane after S_0_→T_1_ excitation with *c* = 22 mM at a) 77 K (*λ*_pump_ = 480 nm), b) 293 K (*λ*_pump_ = 490 nm, time delays of grey spectra 3, 5, 10, 20, and 1000 ps) and after S_0_→S_1_ excitation (*λ*_pump_ = 390 nm) with *c* = 4.8 mM at c) 77 K (time delays of grey spectra 20, 50, 100, 200, 500, and 1000 ps), and at d) 293 K (time delays of grey spectra 3, 5, 10, 20, and 100 ps).

To investigate the photophysical processes on ultrashort time scales, fs‐TA spectroscopy was performed. Pumping the spin‐forbidden S_0_→T_1_ transition required a higher concentration as for S_0_→S_1_ excitation (Figure 8). The TA spectrum of the T_1_ state is immediately observed after S_0_→T_1_ excitation at 77 K (Figure 8a). The observed ESA matches with the weak broad ESA from ns‐TA measurements at 95 K (Figure 7). The ESA narrows with *τ*_1_ = 2.2 ps, which is assigned to VR of the vibrationally hot T_1_ state. The second nondecaying component within the ns regime of the experimental setup obtained from global analysis is ascribed to the vibrationally relaxed T_1_ state. At 293 K, the T_1_ state formed by direct excitation evolves with *τ*_1_ = 14 ps via IC and VR to T_1_’ (Figure 8b). Finally, the equilibrated T_1_/T_1_’ states deactivate to the GS in the ns regime in good agreement to the emission measurements (Figures S18−S21 and Table 1).

The fs dynamics are further investigated probing the spin‐allowed S_0_→S_1_ excitation at 77 K. After fast ISC (≤200 fs instrumental time resolution limit), the ESA of the T_2_ state is observed peaking at ca. 650 nm. The T_2_ ESA then converts to the ESA of T_1_ showing an isosbestic point at 750 nm (Figure 8c). A triexponential fit delivers three time constants with *τ*_1_ = 76 ps, *τ*_2_ = 262 ps and *τ*_3_ for the nondecaying T_1_ state component. *τ*_1_ and *τ*_2_ may be tentatively ascribed to VR and T_2_→T_1_ IC. All these photophysical processes are significantly accelerated in fluid solution at 293 K with *τ*_1_ = 2.6 ps, *τ*_2_ = 5.4 ps and a third nondecaying component for the equilibrated T_1_/T_1_’ states (Figures 8d and S22−S25). The concentration dependence of the T_1_/T_1_’ states’ lifetime is in accordance with the observation of dynamic self‐quenching as discussed above.

**Figure 9:**
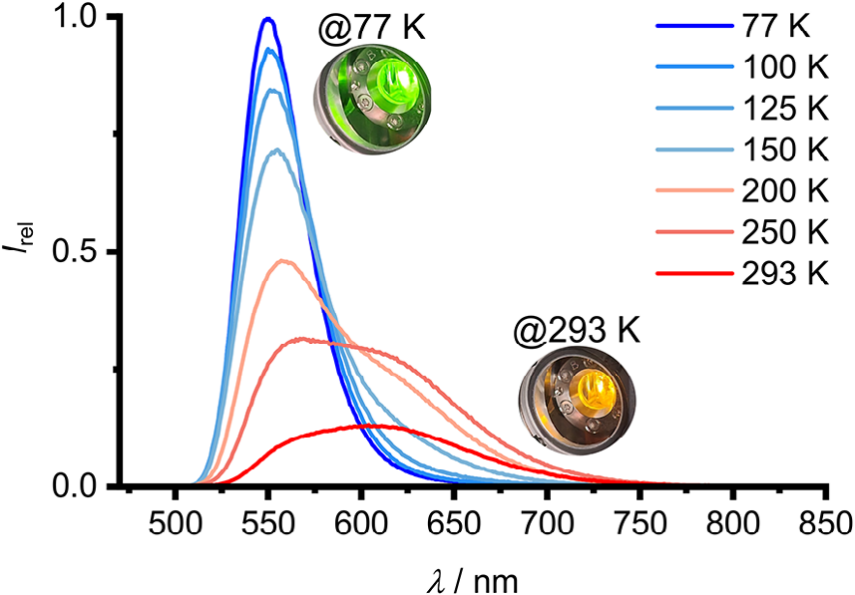
Temperature‐dependent photoluminescence spectra of **Sn** in the solid state between 77 K (blue) and 293 K (red) with *λ*_exc_ = 450 nm (inset: photographs of the green and orange photoluminescence at ca. 77 and 293 K, respectively).

The emission properties of **Sn** in the solid state are very similar to those in solution (Figure 9 and Table 1). The green T_1_→S_0_ phosphorescence is observed over the entire temperature range. The relative intensity of the orange T_1_’→S_0_’ phosphorescence band rises with increasing temperature until 293 K (Figures S26−S29). Interestingly, self‐quenching is effectively prevented in the solid state, leading to very high photoluminescence quantum yields of *Φ* = 0.2 and 0.8 with emission lifetimes *τ* = 8.6 and 21 µs at 293 and 77 K, respectively (Table 1). These numbers outcompete several optimized emitting copper(I) complexes based on thermally activated delayed fluorescence mechanisms.^[^ ^12^, ^13^, ^14^, ^15^ ^]^ The radiative rate constant *k*_r,77_ = 38 000 s^−1^ at 77 K is ascribed to the T_1_ state with a nonradiative rate constant *k*_nr,77_ = 9500 s^−1^. The radiative rate constant *k*_r,293_ = 23 000 s^−1^ at 293 K is smaller with contributions of both triplet states (T_1_ and T_1_’). Therefore, *k*_r_ of the T_1_’ state is smaller than *k*_r_ of the T_1_ state in the solid state. This is also supported by the smaller *k*_r_ = 15 000 s^−1^ determined in solution with dominating T_1_’ state emission. At 293 K in the solid state, thermally activated nonradiative decay is significantly accelerated by one order of magnitude with *k*_nr,293_ = 93 000 s^−1^, which coincides with *k*_nr,0_ = 90 000 s^−1^ obtained from Stern–Volmer analysis in solution. This confirms further, that self‐quenching is negligible in the solid state. The strong and temperature‐dependent green‐orange emission of **Sn** in the solid state (Figure 9) recommends **Sn** as luminescent, time‐gated ratiometric temperature sensor, operating in 150−293 K range by using a single compound and blue light excitation (Boltzmann thermometer; Figure S29).^[^ ^111^, ^112^ ^]^

### Photochemical Properties

Beyond the unique photophysical properties of the unquenched monomeric **Sn** in the solid state and in dilute solution, we studied the self‐quenching of **Sn** and the photoinduced radical formation in solution. With increasing concentration (*c* = 5 − 43 mM, 3‐methylpentane, 77 K), a new weak absorption band appears in the UV–vis absorption spectrum (Figure S30). Hence, we ascribe this band to S_0_^dim^→S_1_^dim^ transitions of loose Van der Waals (VdW) dimers with singlet ground states in the nonpolar hydrocarbon solvent. DFT calculations find two local minima for such loose VdW dimers, namely ***cis*‐^1^[Sn⋯Sn]** and ***trans*‐^1^[Sn⋯Sn]** (Figures S31 and S32). The nature of these VdW dimers and their excimer counterparts will be discussed in more detail below.

**Figure 10:**
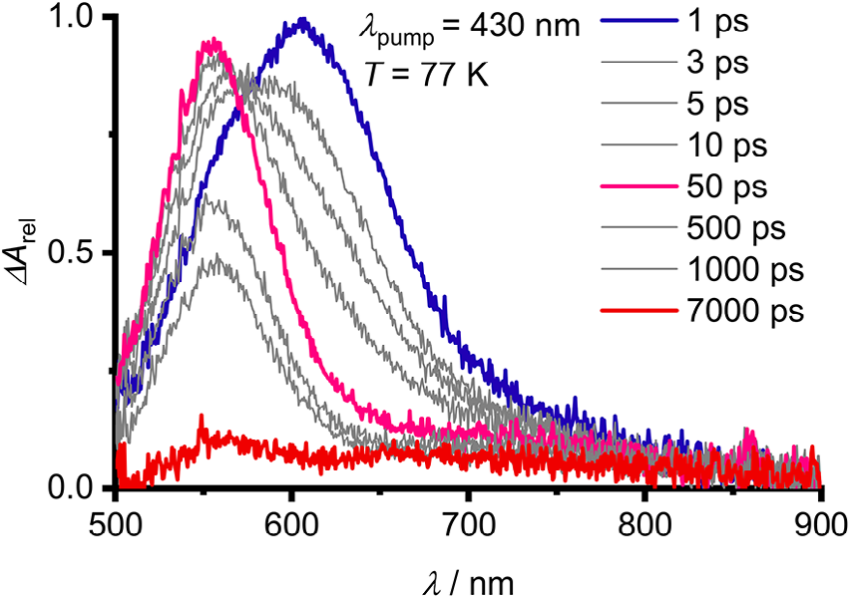
fs‐Transient absorption spectra of **Sn** in 3‐methylpentane at 77 K after excitation at *λ*_pump_ = 430 nm at *c* = 22 mM.

The bathochromic shift of the S_0_^dim^→S_1_^dim^ transitions in comparison to the S_0_→S_1_ transition of **Sn** is supported by TDDFT calculations (Figures 2 and S30−S32). The S_0_^dim^→S_1_^dim^ absorption feature of the VdW dimer is not visible at 293 K, either due to band broadening of the S_0_→S_1_ transition of **Sn** or due to entropy‐driven dissociation of the VdW dimer at higher temperatures (Figure S33). Without emphasizing the TDDFT calculated oscillator strengths too strong, the monomer appears to outnumber the VdW dimers (Figures 2, S3, and S30−S33). To probe the ES dynamics of the VdW dimer, we employed fs‐TA spectroscopy with quite selective S_0_^dim^→S_1_^dim^ excitation (*λ*_pump_ = 430 nm) at 77 K. An ESA at 605 nm is observed (1 ps, Figure 10). With the very similar band position and shape of the T_2_ state ESA of **Sn** (Figures 8c, 10, S34, and S35), we assign this ESA of the VdW dimer to triplet states T_2_^ex^ of the vibrationally hot excimers ***cis***/***trans*‐^3^[Sn⋯Sn]** (1 ps, 605 nm). Consecutive geometry relaxation/cooling by formation of the geometry relaxed excimers ***cis***/***trans*‐^3^[Sn_2_]** in the T_2_^ex^ state occurs with *τ*_1_ = 2.2 ps (Figures 10, S34, and S35). The T_2_^ex^→T_1_^ex^ IC (*τ*_2_ = 11 ps) follows with the ESA of the T_1_^ex^ state peaking at 550 nm. The assignment to T_1_^ex^ states is supported by TDDFT calculations (Figures 10 and S35−S37). Even at 77 K, the thus formed excimers ***cis***/***trans*‐^3^[Sn_2_]** are short‐lived and finally decay with *τ*_3_ = 222 ps and *τ*_4_ = 2.2 ns, respectively (Figures 10 and S34, S35). Under the given excitation conditions, a nondecaying component remains. This corresponds to the long‐lived T_1_ state of the monomeric **Sn**, generated by the direct S_0_→T_1_ excitation via the high‐energy absorption band tail at 430 nm (Figures S38 and S39). Due to broadening of the S_0_→T_1_ absorption band of **Sn** and/or the accelerated excimer deactivation, fs‐TA spectroscopic detection of ***cis***/***trans*‐^3^[Sn_2_]** was impeded at 293 K in fluid solution (Figure S40). Yet, ***cis***/***trans*‐^3^[Sn_2_]** excimer formation is also likely responsible for the dynamic self‐quenching of **Sn** at higher temperature.

**Figure 11:**
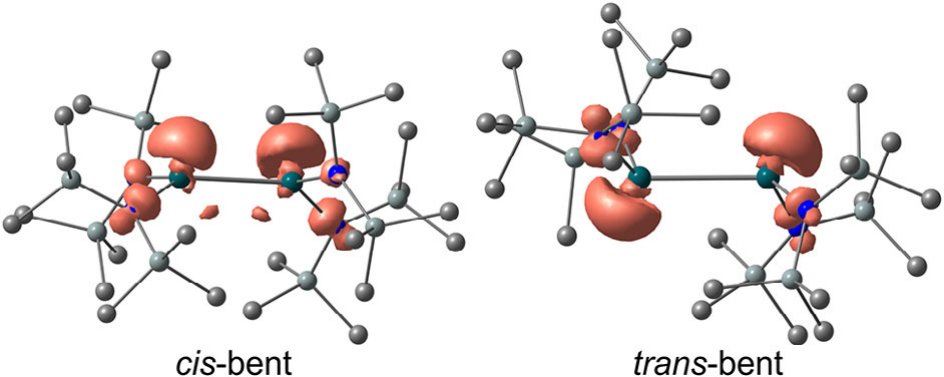
DFT‐calculated optimized geometries of the *cis*‐bent and *trans*‐bent excimers with spin densities (isosurface value: 0.01). CPCM‐(hexane)‐RIJCOSX‐B3LYP‐D3BJ‐SARC/J‐ZORA/def2‐TZVPP/SARC‐ZORA‐TZVPP(Sn)).

DFT geometry optimizations delivered two GS adducts ***cis*‐^1^[Sn⋯Sn]** and ***trans*‐^1^[Sn⋯Sn]** and their respective triplet excimers ***cis*‐^3^[Sn_2_]** and ***trans*‐^3^[Sn_2_]** as local minima with twisted *cis*‐ and *trans*‐bent geometries, respectively (Figures 11 and S41). The spin density in both excimers is mainly located at the tin atoms. The DFT‐derived Sn⋯Sn distances in the GS adducts ***cis*/*trans*‐^1^[Sn⋯Sn]** (3.81 and 3.78 Å) are shorter than those in the XRD structure (4.95 Å)^[^ ^76^ ^]^ with *trans*‐bent geometry (Figure S41). The excimers possess significantly shorter distances of 2.92 and 3.06 Å for ***cis*‐^3^[Sn_2_]** and ***trans*‐^3^[Sn_2_]**, respectively. The *cis* isomers are thermodynamically preferred over their *trans*‐isomer counterparts by Δ*G* = −17 kJ mol^−1^ (−0.18 eV) for ***cis*/*trans*‐^1^[Sn⋯Sn]** and Δ*G* = −27 kJ mol^−1^ (−0.28 eV) for ***cis*/*trans*‐^3^[Sn_2_]**, respectively.

The very small vertical ***cis*‐^3^[Sn_2_]**→ ***cis*‐^1^[Sn_2_]** energy gap of only 0.53 eV (2340 nm) enables very fast nonradiative deactivation (*τ*_3_ = 222 ps, Figure S35) in agreement with the energy‐gap law.^[^ ^11^ ^]^ Thus, excimer formation is responsible for efficient dynamic self‐quenching of the **Sn** phosphorescence in fluid solution by the preferred formation of ***cis*‐^3^[Sn_2_]**. The ***trans*‐^3^[Sn_2_]**→ ***trans*‐^1^[Sn_2_]** vertical energy gap was calculated to 1.33 eV (932 nm), leading to a much slower ES decay (*τ*_4_ = 2.2 ns; Figure S35).

**Figure 12:**
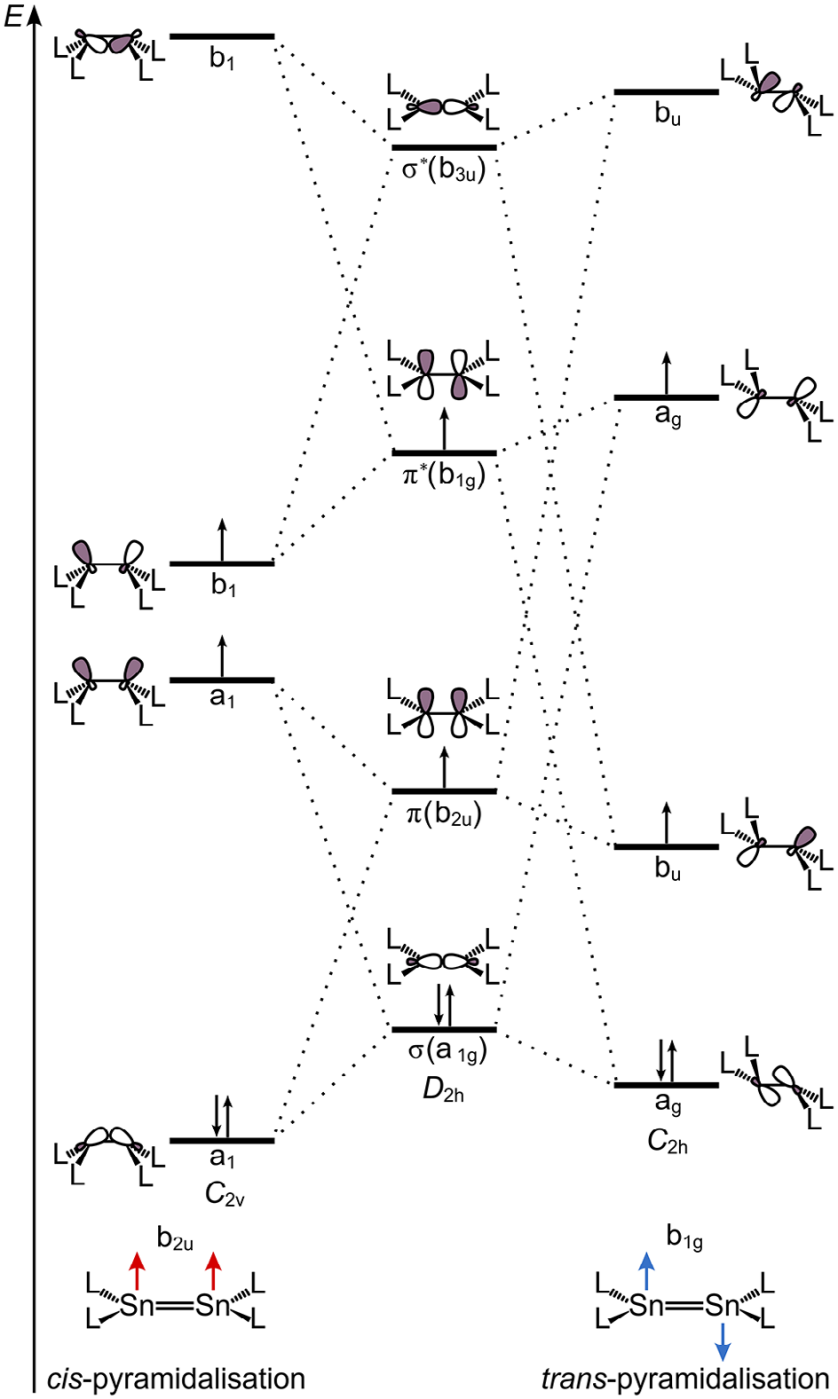
Correlation diagram for the changes in symmetry and energy of frontier orbitals of L_2_Sn = SnL_2_ from planar (*D*_2h_) to *cis*‐bent (*C*_2v_) and *trans*‐bent (*C*_2h_) geometry.

Orbital symmetry considerations help to understand the preferred pyramidalization in the triplet excimers (Figure 12). The *trans*‐bent structure (*C*_2h_) of distannenes in the singlet GS was ascribed to SOJT coupling.^[^ ^44^, ^48^, ^61^, ^62^ ^]^ Analogous *cis*‐ and *trans*‐bent structures for [E_2_L_4_]^•−^ radical anions were also discussed in this context.^[^ ^62^ ^]^ Indeed, *D*_2h_→*C*_2h_ symmetry lowering along the *b*_1g_ vibrational mode mixes the fully occupied *σ*(*a*_g_) and *π*(*b*_2u_) orbitals with the unoccupied π*(*b*_1g_) and σ*(*b*_3u_) orbitals in distannenes in the GS, respectively. The same symmetry argument holds for the triplet state with doubly occupied *σ*(*a*_g_) and singly occupied *π*(*b*_2u_) orbitals, which mix with singly‐ and unoccupied π*(*b*_1g_) and σ*(*b*_3u_) orbitals, respectively, upon *D*_2h_→*C*_2h_ symmetry‐lowering (***trans*‐^3^[Sn_2_]**; Figure 12 and Table S4). On the other hand, the *D*_2h_→*C*_2v_ symmetry change along the *b*_2u_ vibrational mode mixes orbitals, which are closer in energy (*σ*(*a*_g_)↔*π*(*b*_2u_) and π*(*b*_1g_)↔σ*(*b*_3u_)). The resulting smaller orbital energy gap makes the SOJT coupling more effective and hence stabilizes ***cis*‐^3^[Sn_2_]** over ***trans*‐^3^[Sn_2_]**. The *b*_2u_ mode is exclusively SOJT active in the ES, while the *b*_1g_ mode is SOJT active in the GS and ES of distannenes and ethylene homologues in general.

**Figure 13:**
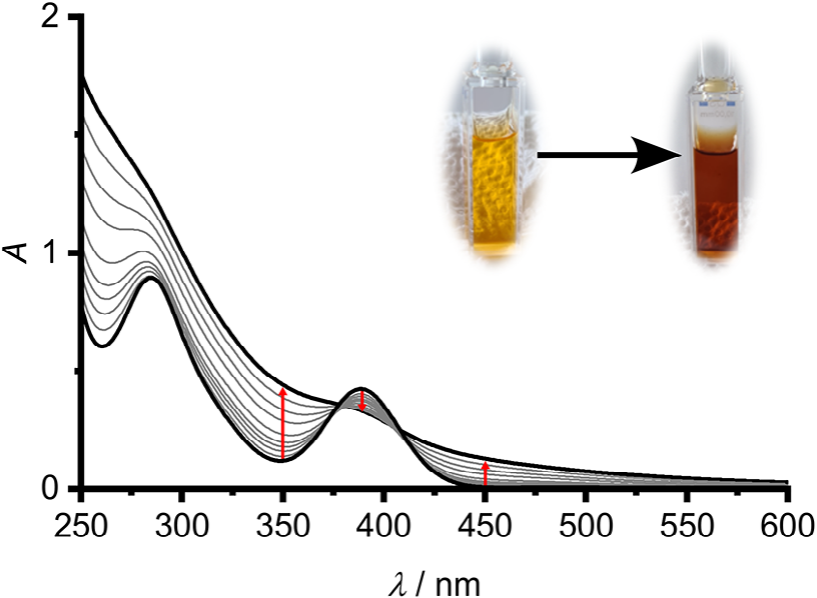
UV–vis absorption spectra (0−15 min) during photolysis of **Sn** at *c* = 0.23 mM in *n*‐pentane irradiated at* λ*_exc_ = 412 nm (*P* = 0.55 W). The photographs show the solution before and after irradiation.

The formation of triplet excimers with concomitant ligand migration might be a step in the photochemical generation of the tin(III) radical •Sn[N(SiMe_3_)_2_]_3_ (mechanism B; Scheme 2b).^[^ ^93^ ^]^ In the bimolecular mechanism B, the rate‐determining step of the radical formation would be the bimolecular excimer formation followed by ligand migration. UV–vis spectroscopic tracking of the photolysis reaction with excitation wavelength *λ*_exc_ = 412 nm and excitation power *P* = 0.55 W, reveals that the color of the solution changes from yellow to brown, without formation of precipitates (Figure 13). During a 15 min period of irradiation, the absorptions of **Sn** almost vanished.

An X‐band EPR spectrum of the resulting brown solution shows the resonance of the expected •Sn[N(SiMe_3_)_2_]_3_ radical (Figure S42).^[^ ^73^, ^92^, ^93^ ^]^ Photolysis experiments at variable concentrations to probe the molecularity of the rate‐determining step reveal a quasi‐linear dependence of absorption versus time within the first 2 min at *λ* = 350 nm (product absorption; Figure S43).

Assuming the concentration of **Sn** as constant (method of initial rates), the photodecomposition yield of *Φ*_deg_ = 0.002 is independent on the concentration and merely depends on the concentration of excited molecules within experimental uncertainty (Figure S43). This clearly supports, as suggested earlier,^[^ ^73^ ^]^ that the •Sn[N(SiMe_3_)_2_]_3_ radical forms following mechanism A via unimolecular tin─ligand bond homolysis of **Sn** (A1) with subsequent •N(SiMe_3_)_2_ radical trapping of **Sn** in the GS (A2) (Scheme 2a). The rate of radical formation is concentration independent, while excimer formation is. This indicates that Sn─N bond homolysis must occur from a short‐lived higher‐lying (hot) ES.

Mechanism A is also preferred based on thermodynamic considerations, with the excimer ***cis*‐^3^[Sn_2_]** being stabilized by 0.11 eV relativeto the formation of the radicals •Sn[N(SiMe_3_)_2_]_3_ and •Sn[N(SiMe_3_)_2_] according to Gibbs free energies from DFT calculations (Scheme 2 and Figure S44). Additionally, the estimated barriers and dissociation limits from 1D and 2D relaxed potential energy surface scans are slightly higher for ligand migration in mechanism B, as compared to ligand dissociation and association in mechanism A (Scheme 2 and Figures S45–S50). Finally, the most striking (kinetic) argument is the very short lifetime of the excimers ***cis*/*trans*‐^3^[Sn_2_]**, preventing any ligand migration before relaxation to the GS.

## Conclusion

The stannylene Sn[N(SiMe_3_)_2_]_2_ **Sn** shows unreported temperature‐dependent dual green/orange phosphorescence with microsecond lifetime from two conformers (T_1_ and T_1_’ states) in solution. At high concentrations, the emission is strongly quenched by triplet excimer formation ***cis*‐** and ***trans*‐^3^[Sn_2_]**. The *cis*‐excimer is thermodynamically preferred by a second‐order Jahn–Teller effect and provides the fastest decay path.

In the solid state, excimer formation is effectively mitigated and very high photoluminescence quantum yields of 0.2 and 0.8 with long microsecond excited state lifetimes are achieved at 293 and 77 K, respectively. These numbers clearly compete with photoluminescent transition metal complexes.

The bimolecular mechanism of the photochemical •Sn[N(SiMe_3_)_2_]_3_ radical generation in solution was ruled out, while the unimolecular Sn─N bond homolysis of Sn[N(SiMe_3_)_2_]_2_ is confirmed as initial rate‐determining step with subsequent aminyl radical trapping by Sn[N(SiMe_3_)_2_]_2_.

The long‐known diamino stannylene Sn[N(SiMe_3_)_2_]_2_ possesses promising photophysical properties—luminescence and long excited state lifetimes. Suppressing excimer and radical formation in suitably designed stannylenes might even pave the way for optical materials and photocatalysis applications. Investigations along these lines are currently in progress.

## Supporting Information

The Supporting Information contains synthesis and separation procedures, experimental spectroscopic data, and quantum chemical data (pdf), including Cartesian coordinates (xyz). The authors have cited additional references within the Supporting Information.^[^ ^52^, ^54^, ^73^, ^113^, ^114^, ^115^, ^116^, ^117^, ^118^, ^119^, ^120^, ^121^, ^122^, ^123^, ^124^, ^125^, ^126^, ^127^, ^128^, ^129^, ^130^, ^131^, ^132^, ^133^, ^134^, ^135^, ^136^, ^137^, ^138^ ^]^

## Conflict of Interests

The authors declare no conflict of interest.

## Supporting information



Supporting Information

Supporting Information

## Data Availability

The data that support the findings of this study are available in the Supporting Information of this article.
