# Supplementary material for: The Overlooked Dual Phosphorescence of Lappert's Diamino Stannylene Sn[N(SiMe3)2]2
Source: Angew Chem Int Ed Engl. 2025 Jul 21;64(36):e202510044. doi: 10.1002/anie.202510044 (PMC12402852; doi:10.1002/anie.202510044)
Supplement: Supplementary file 1 — Supporting Information [file ANIE-64-e202510044-s001.pdf]

Supplementary Information

General

All reactions and measurements were performed under argon atmosphere unless otherwise noted. Gloveboxes (UniLab/MBraun − Ar 4.8, O_2 < 1 ppm, H_2O < 0.1 ppm) were used to store and weigh sensitive compounds for synthesis as well as to prepare samples that require absence of oxygen and water. The reagents were purchased from commercial suppliers (ABCR, Acros Organics, Alfa Aesar, Fischer Scientific and Sigma Aldrich). *n*-pentane and 3-methylpentane were dried and distilled from sodium. C_6D_6 was purchased from euriso-top and Deutero GmbH and was dried and distilled from potassium and stored over molecular sieve (3 Å).

NMR spectra were recorded on a *Bruker Avance NEO* or a *Bruker Avance DRX 400* spectrometer at 400.42 MHz (^{1}H) and 149.23 MHz (^{119}Sn). All data were evaluated with the software *MestReNova 12.0.4-22023*. All resonances are reported in ppm versus the solvent signal as internal standard ^{1}H NMR: C_6D_6: δ = 7.16 ppm[1] or versus an external standard for ^{119}Sn NMR ($SnMe_4$, δ = 0 ppm). (s) = singlet.

UV/Vis absorption spectra were recorded on a *V-770* from *JASCO* or a *Cary-5000* spectrometer from *Agilent* using *d* = 1.00 cm quartz cuvettes with a *Schott* valve. Measurements at low temperatures were carried out using a liquid nitrogen cooled cryostat *Optistat DN* from *Oxford Instruments* equipped with 1 cm quartz tubes. Concentration dependent measurements at low temperatures and room temperature were conducted with a special self-made quartz cell (*d* = 0.2 cm) surrounded with a PEEK frame.

Steady-state emission spectra and photoluminescent decay curves (solution and solid-state samples) were measured with a *FLS1000* spectrometer from *Edinburgh Instruments* equipped with a cooled photomultiplier PMT-980 and a high-speed photomultiplier HSPMT-920 (< 300 ps resolution). A xenon arc lamp Xe2 (450 W) was used for excitation in steady-state measurements. Time-resolved luminescence experiments were conducted using the *µs*-xenon-flashlamp *µF2* (pulse width ca. 2 µs) or a picosecond pulsed diode laser EPL-375 as excitation source. For time resolved emission spectra, a pulsed diode laser VPL-450 was used as excitation source. Measurements at low temperature were carried out using a liquid nitrogen cooled cryostat *Optistat DN* from *Oxford Instruments*. Single lifetime decays were evaluated with the software *Fluoracle* provided by *Edinburgh Instruments*. The data of the time-resolved emission spectra were analysed globally with the open-source *Python* based data analysis tool *KiMoPack 7.4.9*.[2] Absolute luminescence quantum yields Φ were determined using a *MicrostatN* from *Oxford Instruments* combined with a Cryosphere from *Edinburgh Instruments*. Relative uncertainty of Φ is estimated to be ±10%.

Stern-Volmer quenching experiments were carried out in long-neck inert gas quartz cuvettes (*d* = 1.0 cm) with a *Schott* valve. Emission lifetimes and emission spectra were recorded at various concentrations of **Sn** in pentane. The emission lifetimes were measured with a picosecond pulsed diode laser EPL-375 as excitation source. The corresponding emission spectra were recorded upon excitation λ_{exc} = 490 nm ($S_0 \rightarrow T_1$) to guarantee a homogenous excitation in the sample within the concentration range. UV/Vis absorption spectra were measured for each concentration to correct the emission intensities by the respective absorbance.

fs-Transient absorption experiments were conducted using a *Helios* pump-probe setup from *Ultrafast Systems* paired with a regeneratively amplified 1030 nm laser (*Pharos, Light Conversion*, 1030 nm, < 175 fs, 2 mJ). The effective laser repetition rate of 2 kHz was set via an internal pulse picker. A small portion of the 1030 nm fundamental was directed to the optical delay line and was subsequently used to generate broadband probe light by focusing the beam onto a sapphire for measurements in the Vis/NIR range (450 nm – 900 nm). In the UV/Vis spectral range (330 nm – 500 nm), the second harmonic was focused onto a second sapphire. The pump pulse was generated with an optical parametric amplifier (*Apollo Y*, *Ultrafast Systems*) and the beam diameter at the sample was adjusted to 100 µm – 150 µm at the sample to assure homogeneous excitation of the observation volume, which is defined by the probe diameter (ca. 20 µm). At room temperature, the samples were measured under argon atmosphere in a quartz cuvette (*d* = 1 mm). To correct artefacts due to emission and scattering, a second chopper was installed along the probe beam. Measurements at low temperature were carried out using a liquid nitrogen cooled cryostat *Optistat DN* from *Oxford Instruments*. For measurements at

low temperatures, samples were prepared in an air and moisture tight special quartz cell (d = 0.2 cm) surrounded with a PEEK frame (time resolution of ≤ 200 fs for all experiments). Preprocessing of the data, including chirp and baseline correction, has been performed using the *Surface Xplorer 4.3.0* software from *Ultrafast Systems*. The open-source *Python* based data analysis tool *KiMoPack 7.4.9*[2] was employed for global analysis of the TA data.

ns-Transient absorption experiments were carried out using a modified version of the described fs-transient absorption spectroscopy setup. For this purpose, the *EOS* add-on has been employed, which uses a photonic crystal fiber-based supercontinuum laser as probe light source. In contrast to the fs measurements, the pump-probe time delay is controlled electronically with a time resolution of < 1 ns. The sample preparation and data analysis were carried out as described for the fs-transient absorption experiments.

Photolysis experiments were conducted by irradiating deaerated solutions of **Sn** in inert gas cuvettes (d = 1 cm). An Ultra-High Power collimated LED from *Prizmatix* (UHP-T-405-DI) with an emission maximum at 412 nm was used for irradiation. The output power (determined with a high-sensitive thermal power head S425C from *Thorlabs*) was adjusted to P = 0.55 W with an UHPTLCC-02-USB controller for all experiments. The beam was focused onto the cuvette with a plano convex lens. At the cuvette, the beam diameter amounts to 0.5 cm. The temperature of the irradiated solution was kept at 20 °C with a Peltier module.

Irradiation (total irradiation time 15 min) of **Sn** at λ_{exc} = 412 nm in n-pentane leads to tin(III) radical formation, which was followed by UV/Vis absorption spectroscopy and confirmed by EPR spectroscopy. The absorption (A_{350nm}, initial two minutes) versus time plots were fitted linearly for all concentrations (see Figure S43.). Using Lambert-Beer's law, the degradation rates $\dot{N}_{deg}$ were obtained from the slopes $\dot{A}_{350nm}$ of the fits (eq. 1)

$$\frac{dA_{350nm}}{dt} = \dot{A}_{350nm} = \Delta\varepsilon_{350nm} d\dot{c} = \frac{\Delta\varepsilon_{350nm} d}{V N_A} \dot{N}_{deg} \Leftrightarrow \dot{N}_{deg} = \frac{\dot{A}_{350nm} V N_A}{\Delta\varepsilon_{350nm} d} \quad (1)$$

$\Delta\varepsilon_{350nm}$ is the difference between the molar absorption coefficients of the photolysis product and **Sn** at 350 nm, d is the optical path length (1 cm), V is the volume of the sample (3 mL) and N_A is the Avogadro constant.

For a non-monochromatic light source, the rate of photon absorption $\dot{N}_{abs}(\lambda)$ is given by eq. 2.

$$\dot{N}_{abs}(\lambda) = \dot{N}_{LED}(\lambda)\,(1 - 10^{-A(\lambda)}) \quad (2)$$

$\dot{N}_{LED}(\lambda)$ is the rate of photons emitted at the corresponding wavelength and is obtained from the emission spectrum of the UHP-LED, which is multiplied with the photon energy $\frac{h\,c}{\lambda}$ at the respective wavelength. The spectral area is approximated as sum of intensities in 0.5 nm steps and normalised to the output power of P = 0.55 W. The number of emitted photons is obtained from division by $\frac{h\,c}{\lambda}$. $A(\lambda)$ is the initial absorbance of the sample and is assumed to be constant for the first two minutes. The total rate of photon absorption $\dot{N}_{abs}$ is is given by the sum of all $\dot{N}_{abs}(\lambda)$ in the wavelength range 370 – 480 nm (eq. 3).

$$\dot{N}_{abs} = \sum_{\lambda} \dot{N}_{abs}(\lambda) \quad (3)$$

The photodegradation quantum yield Φ_{deg} is defined as the ratio between the number of degraded molecules N_{deg} and the total number of absorbed photons N_{abs}, which equals with the ratio between the degradation rate $\dot{N}_{deg}$ of the chromophore and the total rate of photon absorption $\dot{N}_{abs}$ (eq. 4).

$$\Phi_{deg} = \frac{N_{deg}}{N_{abs}} = \frac{\dot{N}_{deg}}{\dot{N}_{abs}} \quad (4)$$

X-band cw-electron paramagnetic resonance measurements were carried out using a *Magnettech Miniscope MS 300* in 3-methylpentane at 293 K. *g* values are referenced to external Mn^{2+} in ZnS (*g* = 2.118(1), 2.066(1), 2.027(2), 1.986(2), 1.946(1), 1.906(1)). Microwave frequency: 9.427917 GHz, center field: 338 mT, sweep: 20 mT, modulation amplitude 0.2 mT, receiver gain = 10, microwave attenuation 10 dB, sweep time: 90 s. Simulations of the experimental spectra were performed with *garlic* (isotropic, fast-motional cw-EPR spectra) of *EasySpin* (5.2.35) for *Matlab R2020a*.[3]

Density functional theory calculations were performed with *Orca 5.0.4*.[4,5] Geometry optimisation was performed using (un-)restricted Kohn-Sham orbitals DFT (UKS/RKS) and the B3LYP functional[6–8] in combination with Ahlrich's split valance triple-zeta basis set def2-TZVPP[9] and SARC-ZORA-TZVPP[10] (Sn) with the auxiliary basis SARC/J[11–14]. Tight convergence criteria were chosen for DFT calculations (keywords TIGHTSCF and TIGHTOPT). All DFT calculations make use of the resolution of identity (Split-RI-J) approach for the Coulomb term in combination with the chain-of-spheres approximation for the exchange term (keyword RIJCOSX[15,16]). The zeroth order regular approximation was used to describe relativistic effects in all calculations (keyword ZORA).[11–14,17,18] To account for solvent effects, a conductor-like screening model (keyword CPCM(hexane)) modelling hexane was used in all calculations.[19,20] Atom-pairwise dispersion correction was performed with the Becke-Johnson damping scheme (keyword D3BJ[21,22]). A numerical frequency calculation confirmed that the optimised geometry corresponds to a minimum structure or a transition state structure, respectively. The 2D relaxed potential energy surface scans with 72 points were performed on the CPCM(hexane)-RIJCOSX-(U)B3LYP-D3BJ-def2/J-def2-SVP level with subsequent single-point calculations at higher level (CPCM(hexane)-RIJCOSX-(U)B3LYP-D3BJ-SARC/J-ZORA/def2-TZVPP/SARC-ZORA-TZVPP(Sn)). Transition states were localised, using the OPTTS keyword and assigned with a subsequent calculation of the internal reaction coordinate (keyword IRC). Fifty spin-allowed transitions were calculated by TDDFT. Spin Orbit Coupling (SOC) TDDFT calculations were performed using the additional keyword RI-SOMF(1X).[23] Twenty vertical spin-forbidden transitions were localised with TRIPLETS TRUE as keyword. The charge transfer number analyses of the TDDFT calculated transitions were done using *TheoDORE 2.4*.[24] All calculations were computed on the supercomputer Elwetritsch and advisory services offered by the RPTU Kaiserslautern-Landau (https://hpc.rz.rptu.de); which is a member of the AHRP. All coordinates are provided in an additional xyz file.

I Synthesis

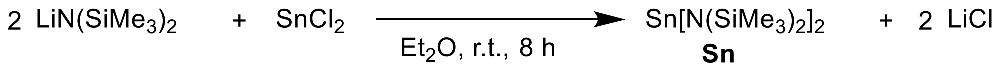

$Sn[N(SiMe_3)_2]_2$ (Sn): Sn was prepared according to modified literature procedures.[25–27] In a Schlenk tube, lithium bis(trimethylsilyl)amide (21.20 g, 127 mmol, 2 eq) was combined with tin dichloride (12.00 g, 63 mmol, 1 eq). Diethylether (300 mL) was added slowly under strong stirring and cooling (ice-water bath). Immediately, the cloudy colourless mixture turned orange and the reaction was stirred for 8 h at room temperature. The resulting LiCl was removed from the solution by filtration and the solvent was removed under reduced pressure resulting an orange oil. The crude product was distilled under reduced pressure leading the desired product as a red-orange oil, which solidified with liquid nitrogen cooling to a yellow-orange solid. (20.22 g, 46 mmol, 73%), ^{1}H NMR (400 MHz, C_6D_6): δ = 0.29 (s, CH_3) ppm. ^{119}Sn NMR (149 MHz, C_6D_6): 766.6 ppm. UV/Vis (*n*-pentane): λ (ε) = 490 (31), 389 (1830), 287 nm (3080 M^{-1} cm^{-1}). The data match literature values.[27–29]

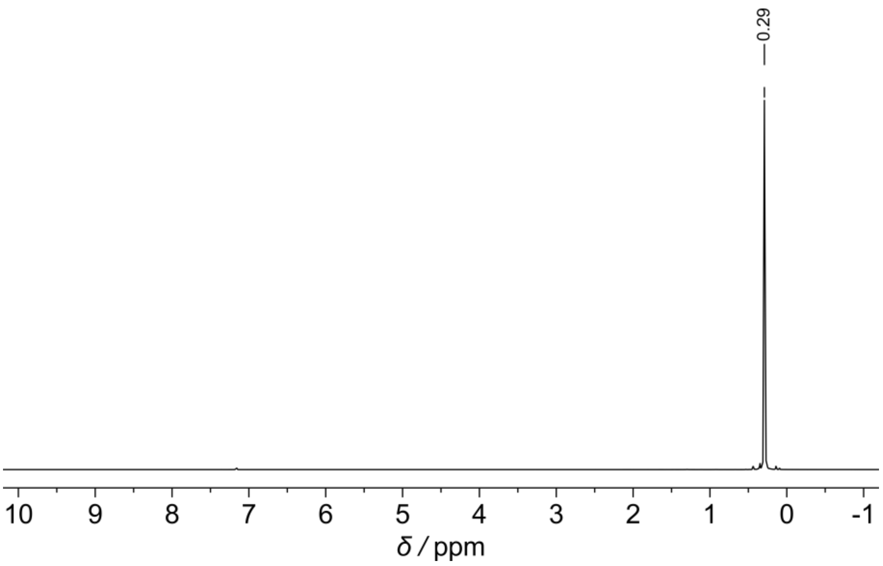

Figure S1. ^{1}H NMR spectrum of **Sn** in C_6D_6.

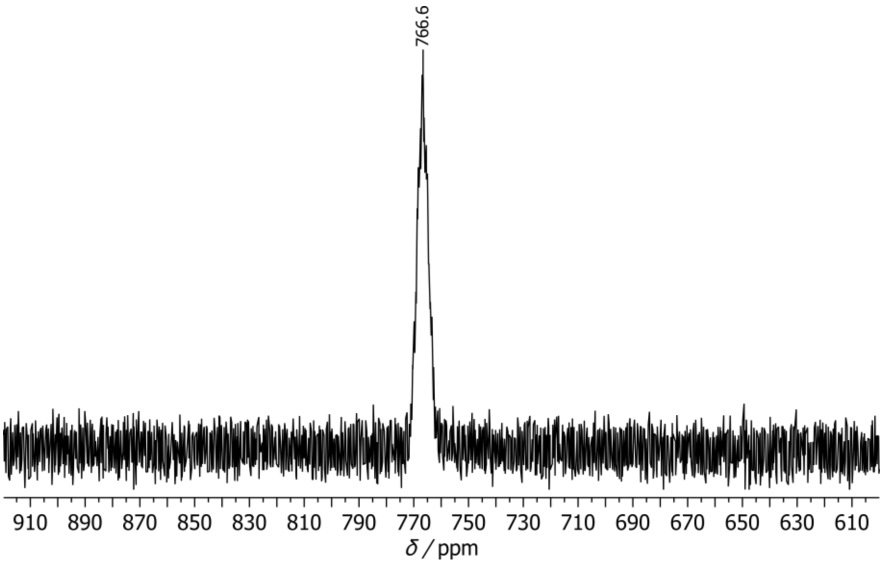

Figure S2. ^{119}Sn NMR spectrum of **Sn** in C_6D_6.

II Photophysical Properties

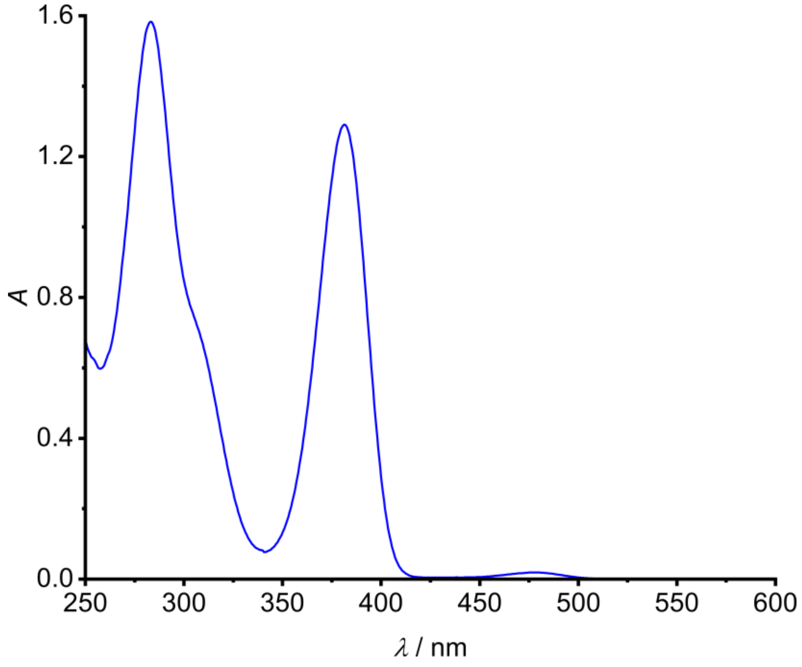

Fig. S3. UV/Vis absorption spectrum of **Sn** (*c* = 0.36 mM) in 3-methylpentane at 77 K.

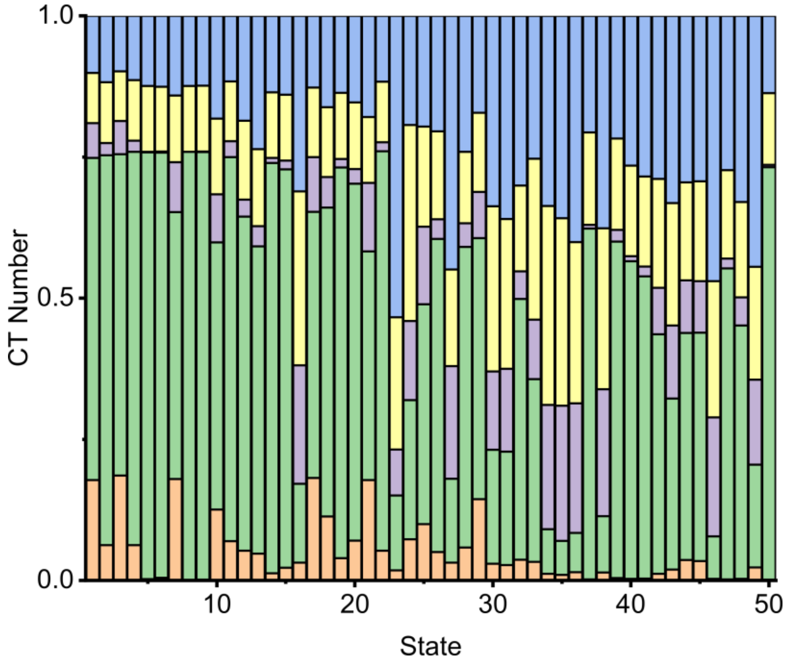

Figure S4. Charge transfer number analysis of the 50 lowest spin-allowed transitions of **Sn** (orange: MC, green: LMCT, purple: MLCT; yellow: LLCT, blue: LC). CPCM(hexane)-RIJCOSX-B3LYP-D3BJ-SARC/J-ZORA/def2-TZVPP/SARC-ZORA-TZVPP(Sn)).

Table S1. Selected molecular orbitals of **Sn** with energies given in eV, displayed at an isosurface value 0.06 a.u.. Mulliken symbol of the orbital character given in idealised C_{2v} point group of the N-Sn-N unit. Hydrogen atoms omitted. CPCM(hexane)-RIJCOSX-B3LYP-D3BJ-SARC/J-ZORA/def2-TZVPP/SARC-ZORA-TZVPP(Sn)).

HOMO−2(a_2) / −6.8171	HOMO−1(b_2) / −6.4389	HOMO(a_1) / −5.9203	LUMO(b_2) / −1.9977
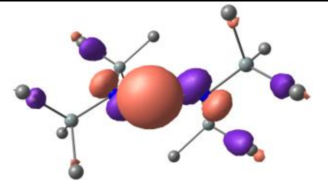	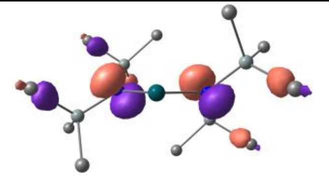	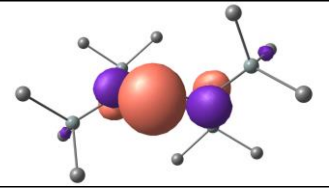	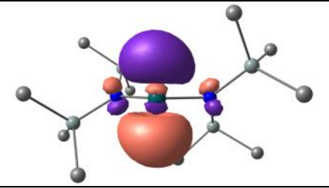

Table S2. SOC constants as square root of the sum of the squares of SOC matrix elements in cm^{-1} between the lowest singlet and triplet states derived from SOC-TDDFT calculations on the geometry optimised GS structure of **Sn**. CPCM(hexane)-RIJCOSX-B3LYP-D3BJ-SARC/J-ZORA/def2-TZVPP/SARC-ZORA-TZVPP(Sn)).

**triplet states**	**singlet states**			
	**S_0**	**S_1**	**S_2**	**S_3**
**T_1**	945	94	282	120
**T_2**	63	194	48	112
**T_3**	654	15	224	8

Table S3. SOC corrected TDDFT transitions of **Sn** with energies E in cm^{-1} and wavelengths λ in nm and oscillator strengths f. CPCM(hexane)-RIJCOSX-B3LYP-D3BJ-SARC/J-ZORA/def2-TZVPP/SARC-ZORA-TZVPP(Sn)).

**State**	Energy / cm^{-1}	λ / nm	f
**S_1**	24927	401.2	0.033964539
**S_2**	29745	336.2	0.013170607
**S_3**	32309	309.5	0.059986636
**T_1**	19510	512.6	0.000000300
	19534	511.9	0.000273717
	19569	511.0	0.002700203
**T_2**	26223	381.3	0.000000473
	26228	381.3	0.000005283
	26259	380.8	0.001629286
**T_3**	28386	352.3	0.003692885
	28402	352.1	0.000013474
	28412	352.0	0.000021312

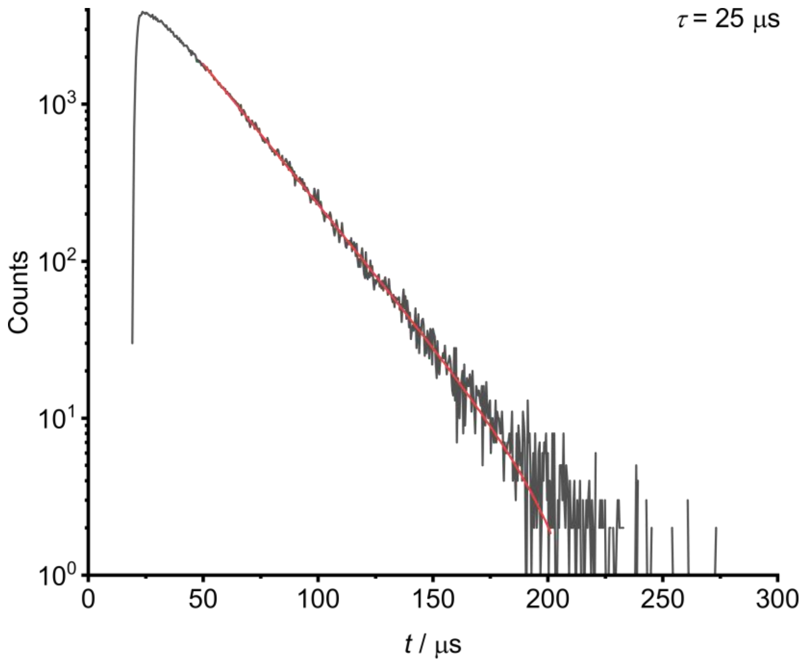

Figure S5. Emission decay curve of **Sn** in 3-methylpentane at λ_{em} = 560 nm upon λ_{exc} = 390 nm excitation (pulse width = 2 μs) at 77 K superimposed with monoexponential fit (red curve).

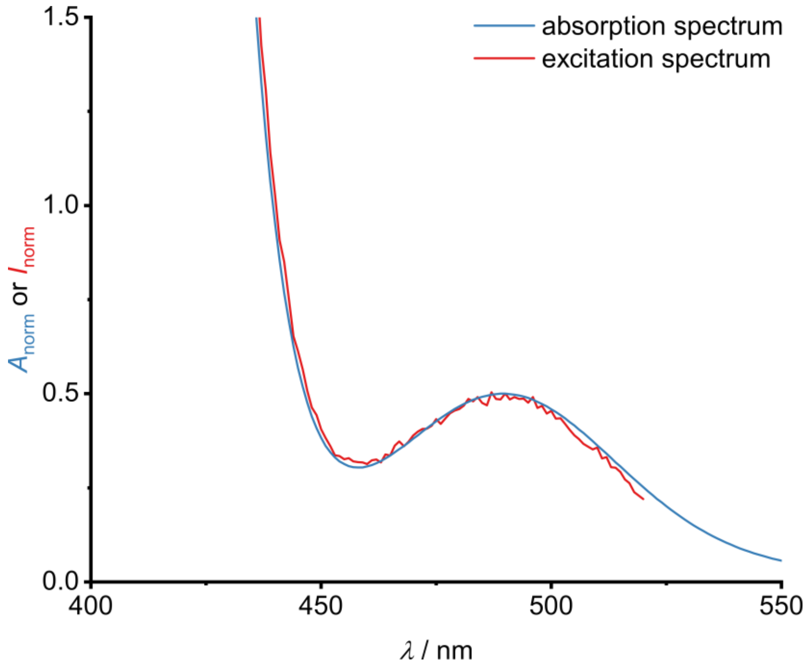

Figure S6. Excitation spectrum of **Sn** in 3-methylpentane at 293 K (λ_{em} = 640 nm c = 2.5 mM, red) and UV/Vis absorption spectrum of **Sn** in 3-methylpentane at 293 K (c = 1.7 mM, blue).

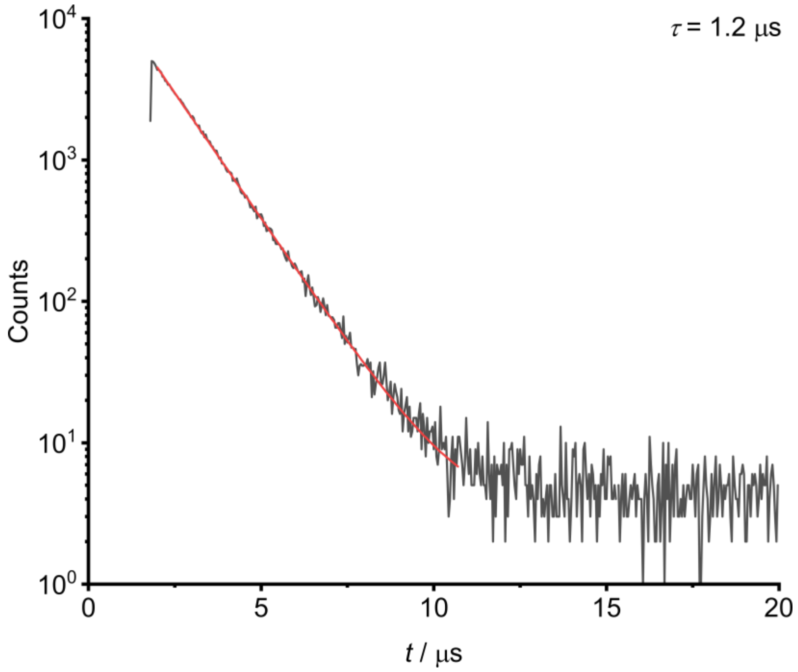

Figure S7. Emission decay curve of **Sn** in *n*-pentane upon λ_{exc} = 375 nm excitation at λ_{em} = 640 nm at 293 K superimposed with monoexponential fit (red curve) (c = 0.32 mM).

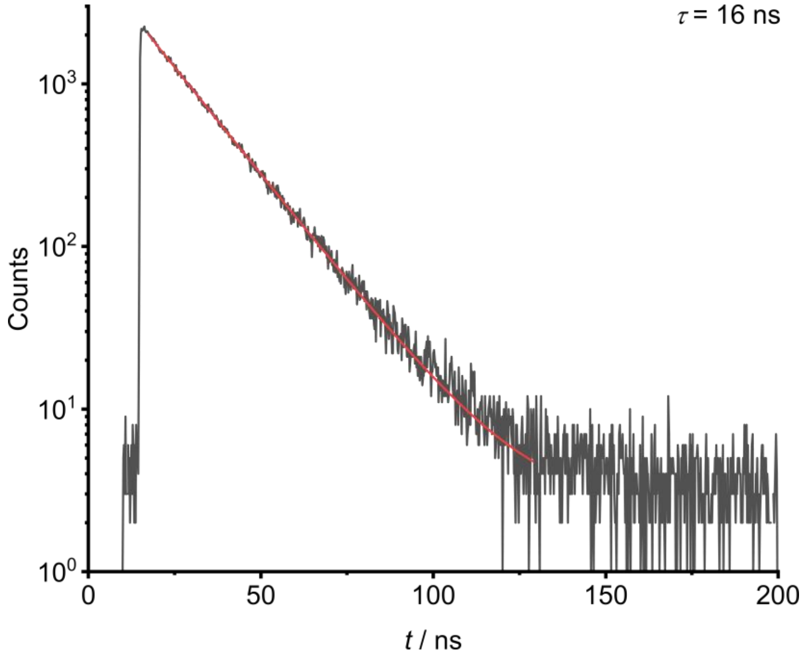

Figure S8. Emission decay curve of **Sn** in *n*-pentane upon λ_{exc} = 375 nm excitation at λ_{em} = 640 nm at 293 K superimposed with monoexponential fit (red curve) (c = 25 mM).

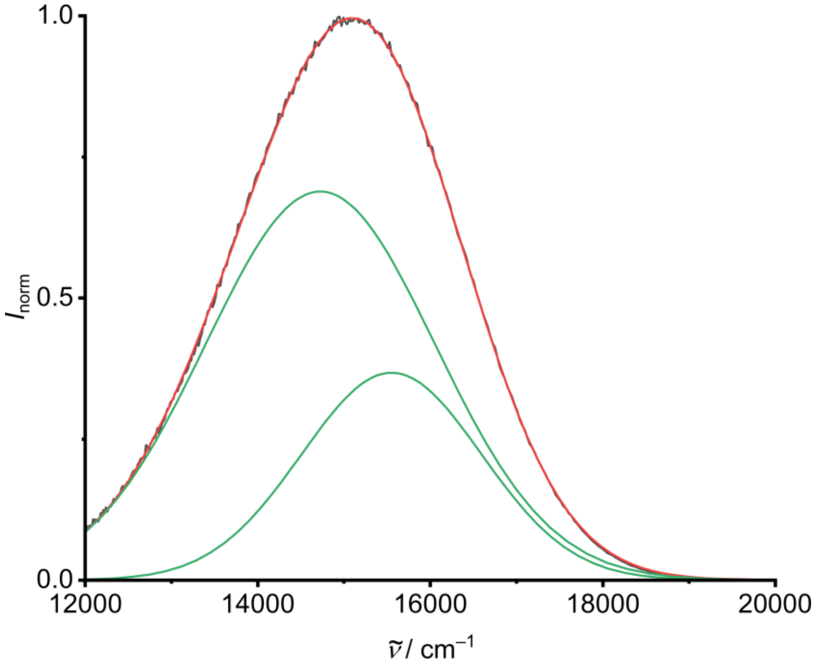

Type	Area	FWHM / cm^{-1}	Max Height	Centre / cm^{-1}	Area ratio / %
Voigt	2268	3134	0.69	14725	70
Voigt	961	2459	0.37	15552	30

Figure S9. Emission spectrum of **Sn** in 3-methylpentane at 293 K in black overlayed with two Voigt functions. Fit components shown in green and the cumulative fit in red with fit parameters.

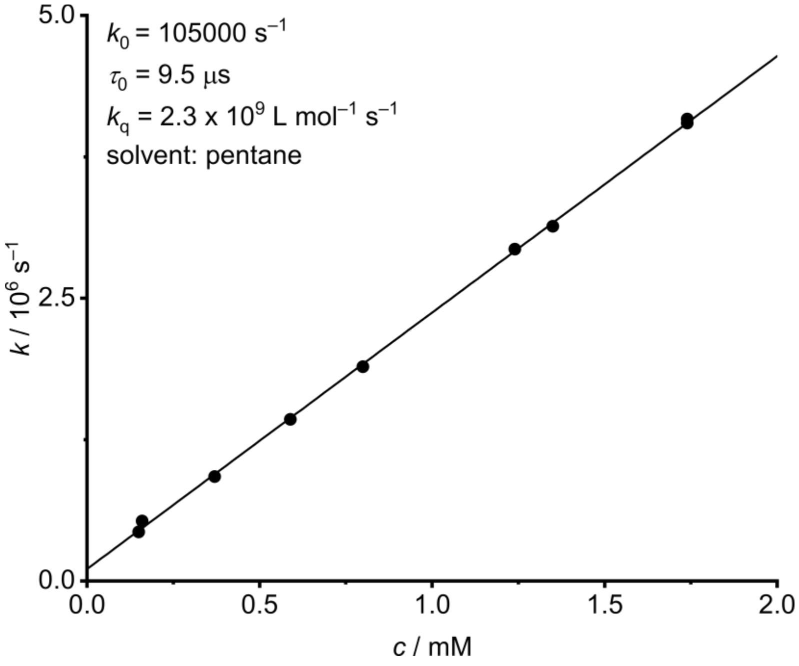

Figure S10. Modified Stern-Volmer plot of quenching rate constants of **Sn** in *n*-pentane upon λ_{exc} = 375 nm excitation ($S_0 \rightarrow S_1$) with λ_{em} = 640 nm at 293 K at various concentrations with linear fit.

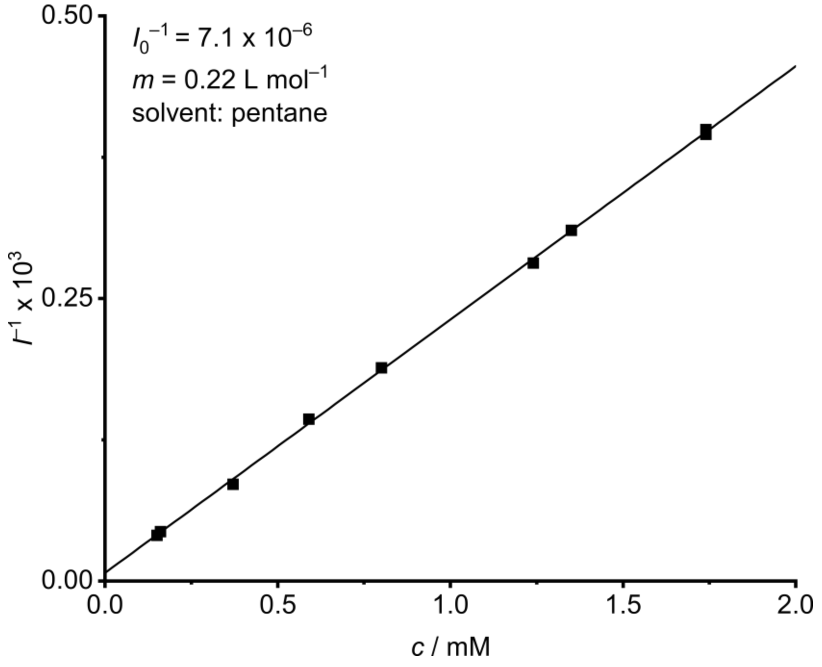

Figure S11. Modified Stern-Volmer plot of the reciprocal emission intensity from absorption corrected integration of **Sn** in n-pentane, emission upon λ_{exc} = 490 nm excitation ($S_0 \rightarrow T_1$, to omit self-absorption) at 293 K at various concentrations with linear fit.

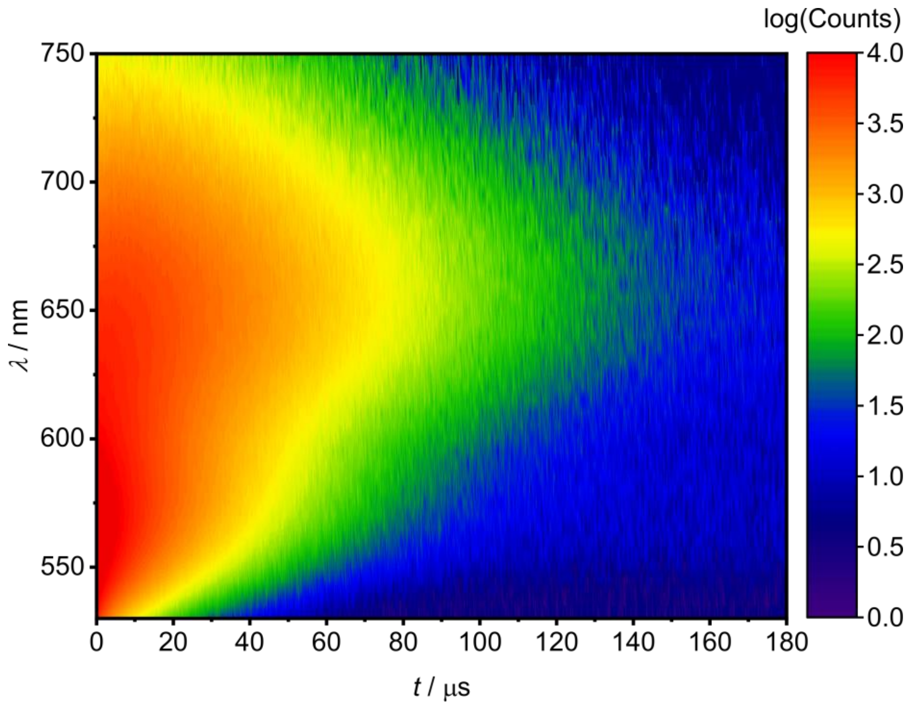

Fig. S12. Colour map of the time-resolved emission spectra of **Sn** in 3-methylpentane obtained upon λ_{exc} = 450 nm laser excitation at 95 K.

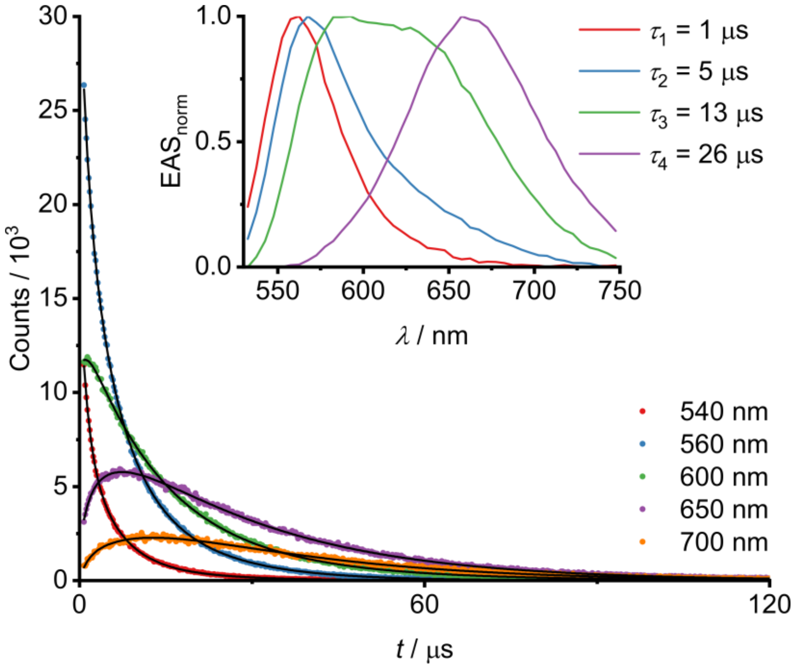

Figure S13. Emission decay curves of **Sn** in 3-methylpentane upon λ_{exc} = 450 nm laser excitation at 95 K at selected wavelengths with multiexponential fits obtained by global analysis (black curve, τ_1 = 1 µs, τ_2 = 5 µs, τ_3 = 13 µs, τ_4 = 26 µs, sequential model). Inset: Evolution associated spectra acquired from global analysis.

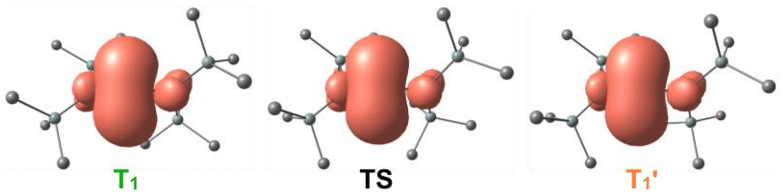

Figure S14. DFT calculated optimised geometries of the triplet states T_1 and T_1' and the $T_1 \leftrightarrow T_1$' transition state of **Sn** with spin densities (isosurface value 0.005 a.u.). Hydrogen atoms omitted. CPCM(hexane)-RIJCOSX-B3LYP-D3BJ-SARC/J-ZORA/def2-TZVPP/SARC-ZORA-TZVPP(Sn)).

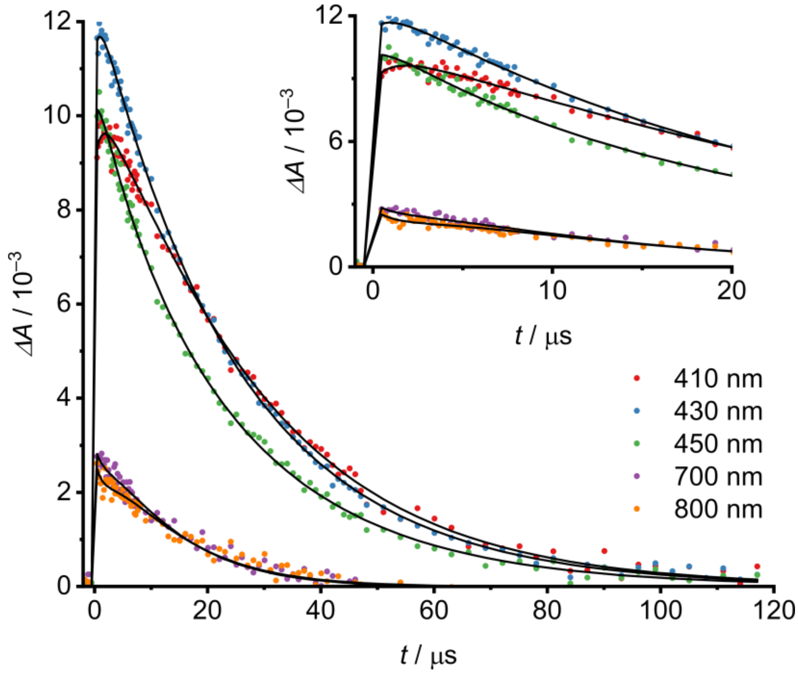

Figure S15. TA decay curves of **Sn** in 3-methylpentane upon λ_{pump} = 390 nm excitation at 95 K at selected wavelengths with multiexponential fits obtained from global analysis (black curves, τ_1 = 1 µs, τ_2 = 5 µs, τ_3 = 13 µs, τ_4 = 26 µs, sequential model). The inset shows a zoom in the 0−20 µs range.

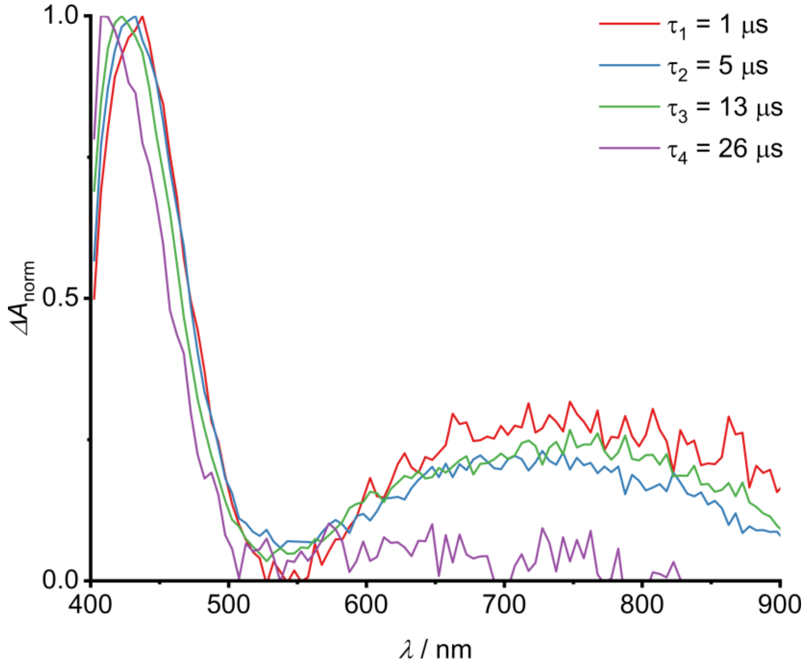

Figure S16. Evolution associated difference absorption spectra of **Sn** in 3-methylpentane upon λ_{pump} = 390 nm excitation at 95 K obtained from global analysis with corresponding lifetimes of the respective components.

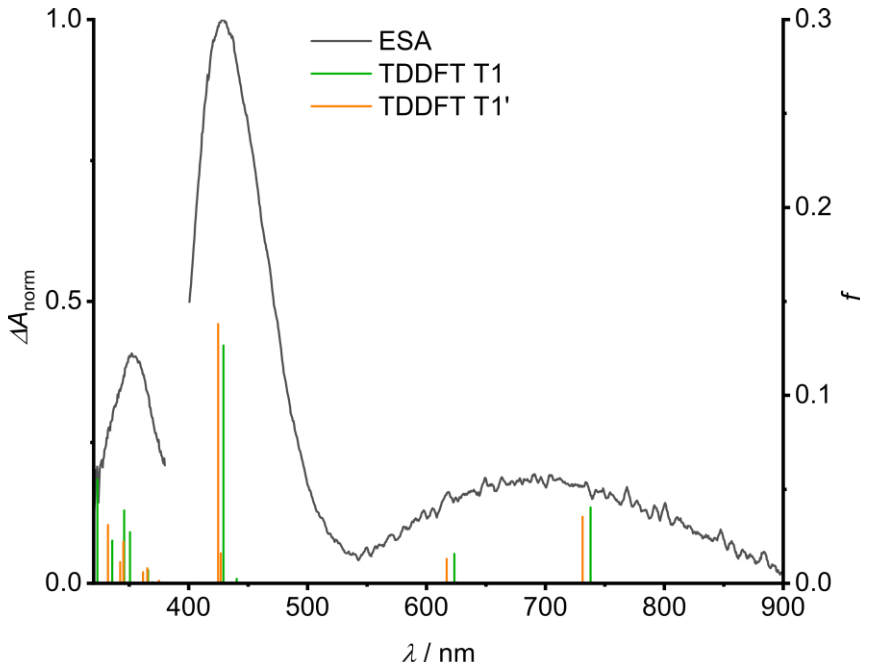

Figure S17. Normalised, time-averaged (5–40 ns) excited state absorption (ESA) spectrum of **Sn** in 3-methylpentane (c = 4.8 mM) at 293 K after $S_0 \rightarrow S_1$ excitation at λ_{pump} = 390 nm with TDDFT calculated oscillator strengths of T_1 and T_1', shifted by 0.5 eV to higher energies, at the relaxed triplet geometries in green and orange, respectively. CPCM(hexane)-RIJCOSX-B3LYP-D3BJ-SARC/J-ZORA/def2-TZVPP/SARC-ZORA-TZVPP(Sn)).

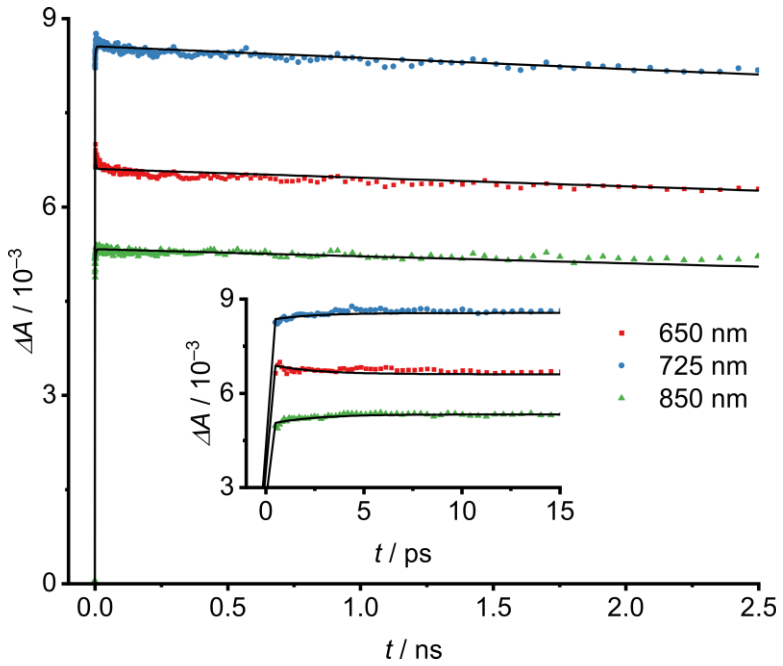

FigureS18. TA decay curves of **Sn** in 3-methylpentane at 77 K upon $S_0 \rightarrow T_1$ λ_{pump} = 480 nm excitation at selected wavelengths with biexponential fits obtained from global analysis (black curves τ_1 = 2.2 ps, τ_2 = non decaying, sequential model). The inset shows a zoom in the 0−15 ps range.

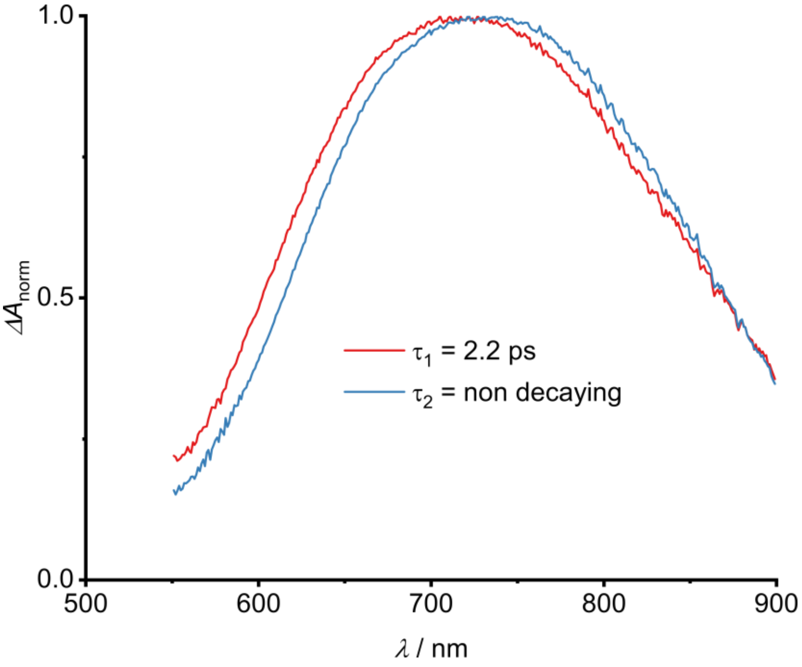

Figure S19. Evolution associated difference absorption spectra of **Sn** in 3-methylpentane at 77 K after $S_0 \rightarrow T_1$ excitation at λ_{pump} = 480 nm obtained from global analysis with corresponding lifetimes of the respective components.

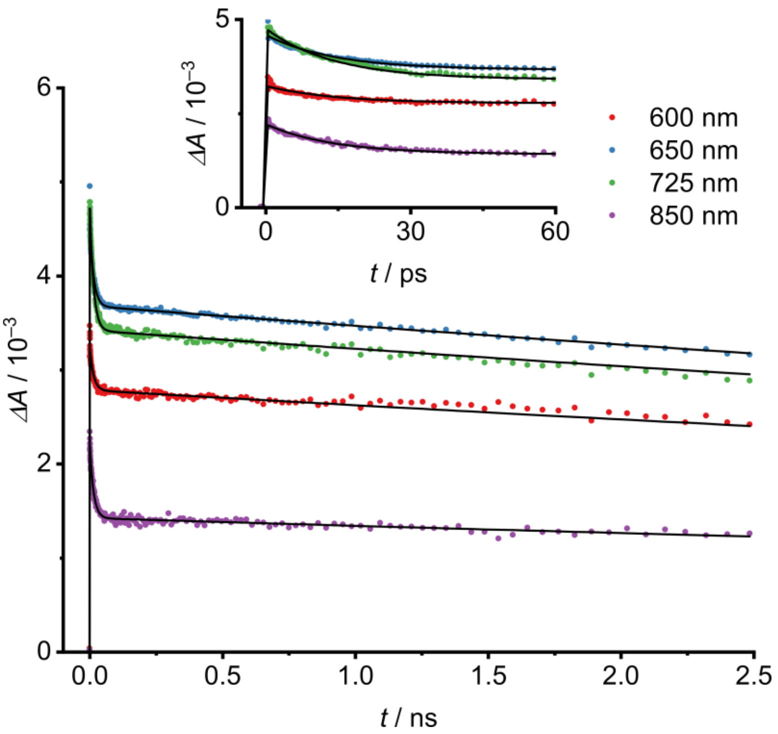

Figure S20. TA decay curves of **Sn** in 3-methylpentane at 293 K upon $S_0 \rightarrow T_1$ λ_{pump} = 490 nm excitation at selected wavelengths with biexponential fits obtained from global analysis (black curves, τ_1 = 14 ps, τ_2 = non decaying (17 ns), sequential model). The inset shows a zoom in the 0−60 ps range.

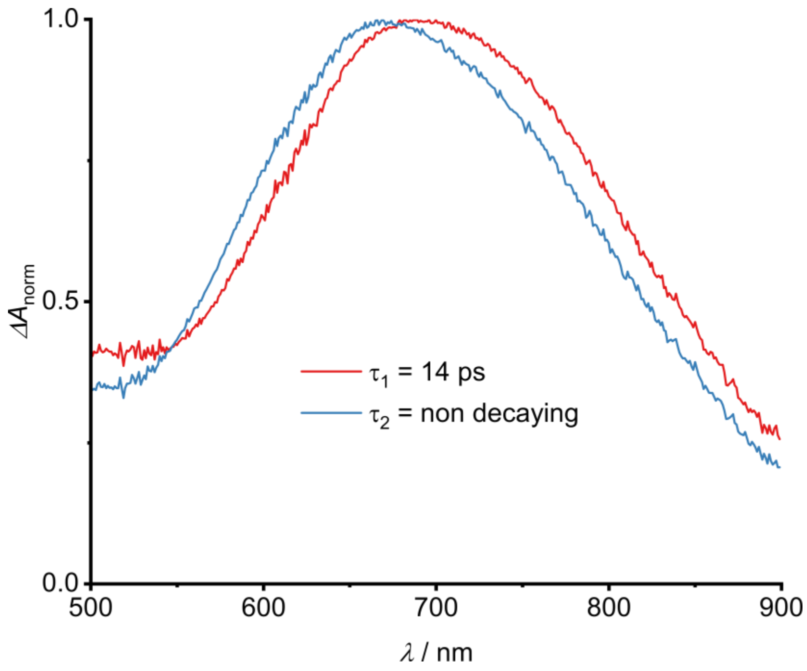

Figure S21. Evolution associated difference absorption spectra of **Sn** in 3-methylpentane at 293 K after $S_0 \rightarrow T_1$ excitation at λ_{pump} = 490 nm obtained from global analysis with corresponding lifetimes of the respective components.

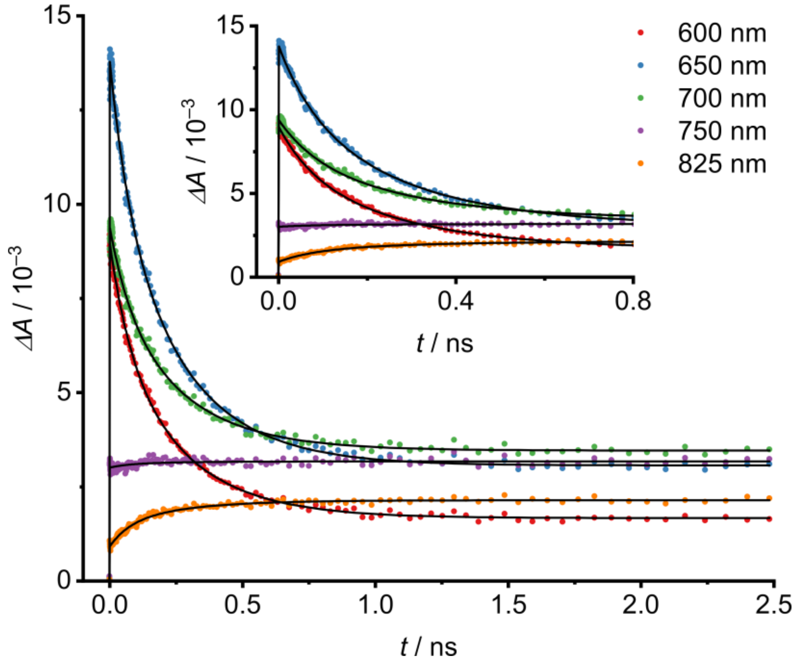

Figure S22. TA decay curves of **Sn** in 3-methylpentane at 77 K upon $S_0 \rightarrow S_1$ λ_{pump} = 390 nm excitation at selected wavelengths with triexponential fits obtained from global analysis (black curves, τ_1 = 76 ps, τ_2 = 262 ps, τ_3 = non decaying, sequential model). The inset shows a zoom in the 0−0.8 ns range.

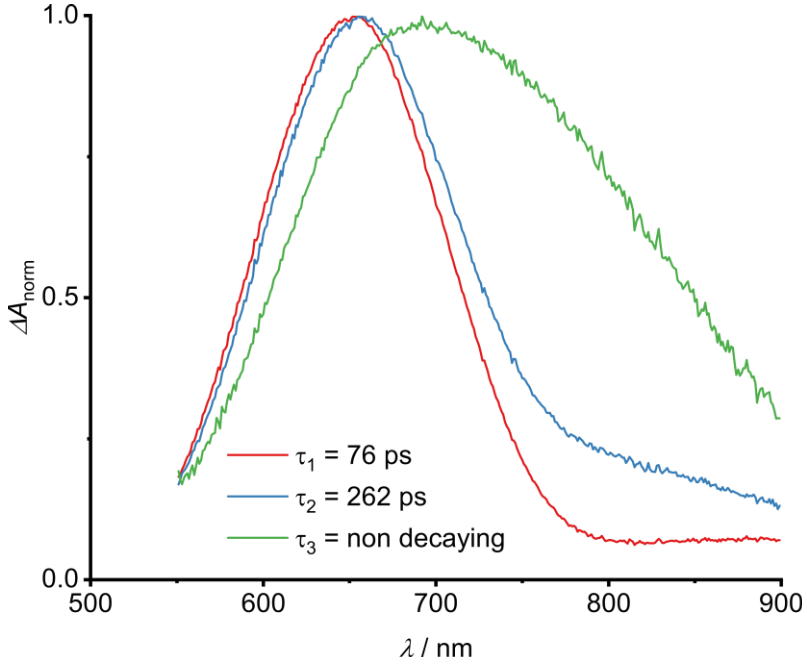

Figure S23. Evolution associated difference absorption spectra of **Sn** in 3-methylpentane at 77 K after $S_0 \rightarrow S_1$ excitation at λ_{pump} = 390 nm obtained from global analysis with corresponding lifetimes of the respective components.

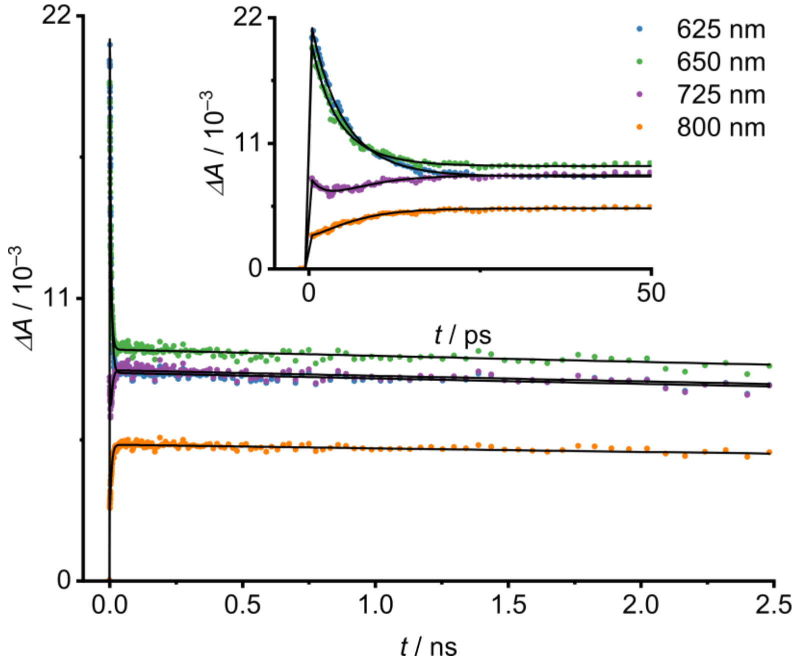

Figure S24. TA decay curves of **Sn** in 3-methylpentane at 293 K upon $S_0 \rightarrow S_1$ λ_{pump} = 390 nm excitation at selected wavelengths with triexponential fits obtained from global analysis (black curves, τ_1 = 2.6 ps, τ_2 = 5.4 ps, τ_3 = non decaying, sequential model). The inset shows a zoom in the 0−50 ps range.

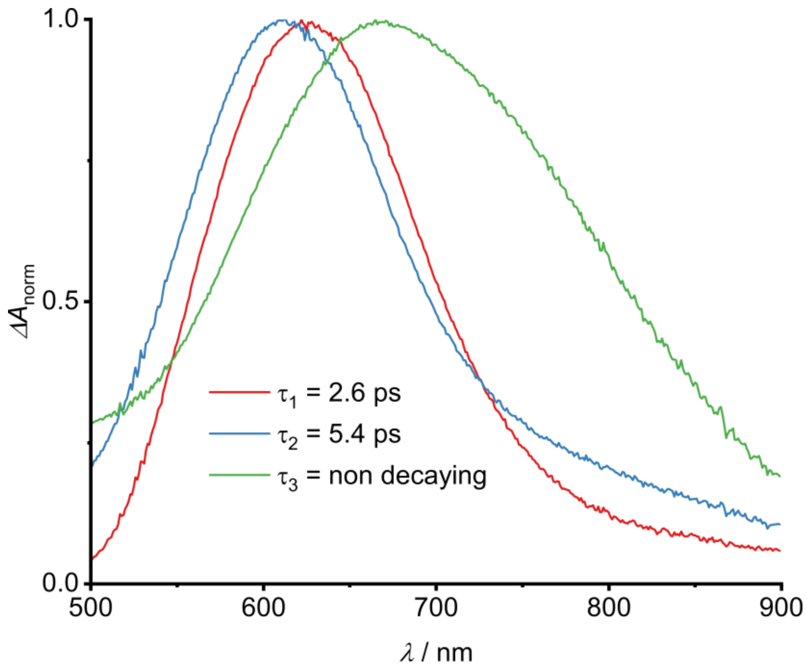

Figure S25. Evolution associated difference absorption spectra of **Sn** in 3-methylpentane at 293 K after $S_0 \rightarrow S_1$ excitation at λ_{pump} = 390 nm obtained from global analysis with corresponding lifetimes of the respective components.

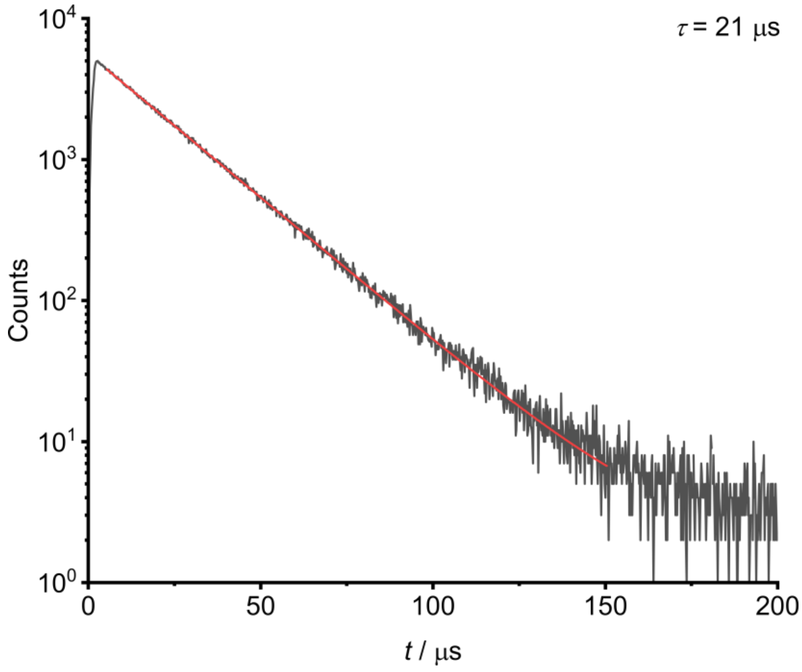

Figure S26. Emission decay curve of **Sn** (solid) at 77 K at λ_{em} = 550 nm upon $S_0 \rightarrow T_1$ λ_{exc} = 450 nm excitation superimposed with monoexponential fit (red curve).

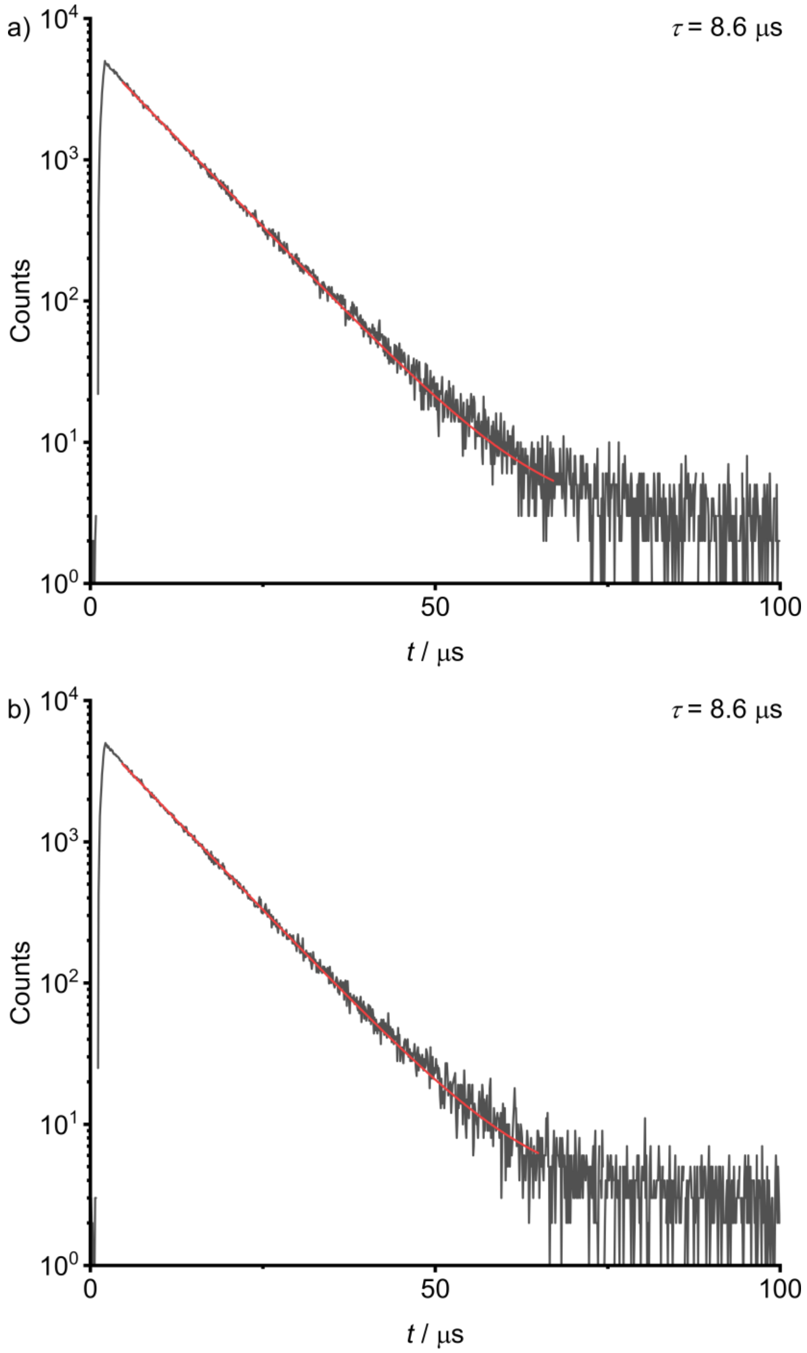

Figure S27. Emission decay curves of **Sn** (solid) at 293 K at a) λ_{em} = 550 nm upon $S_0 \rightarrow T_1$ λ_{exc} = 450 nm excitation and b) λ_{em} = 650 nm upon $S_0 \rightarrow T_1$ λ_{exc} = 450 nm excitation superimposed with monoexponential fits (red curves).

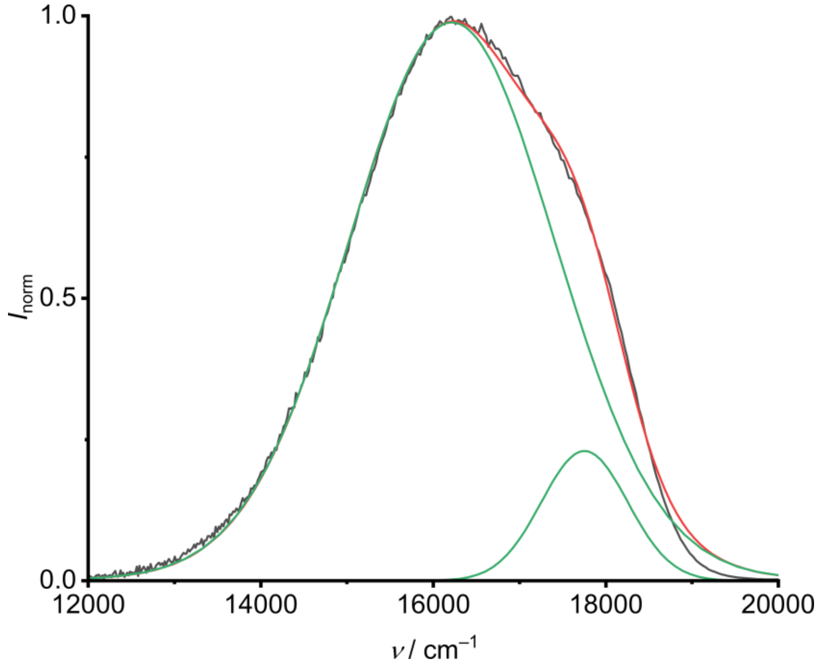

Type	Area	FWHM / cm^{-1}	Max Height	Centre / cm^{-1}	Area ratio / %
Voigt	2982	2820	0.99	16216	91
Voigt	288	1179	0.23	17754	9

Figure S28. Emission spectrum of **Sn** (solid) upon $S_0 \rightarrow T_1$ λ_{exc} = 450 nm excitation at 293 K in black overlayed with two Voigt functions. Fit components shown in green and the cumulative fit in red with fit parameters.

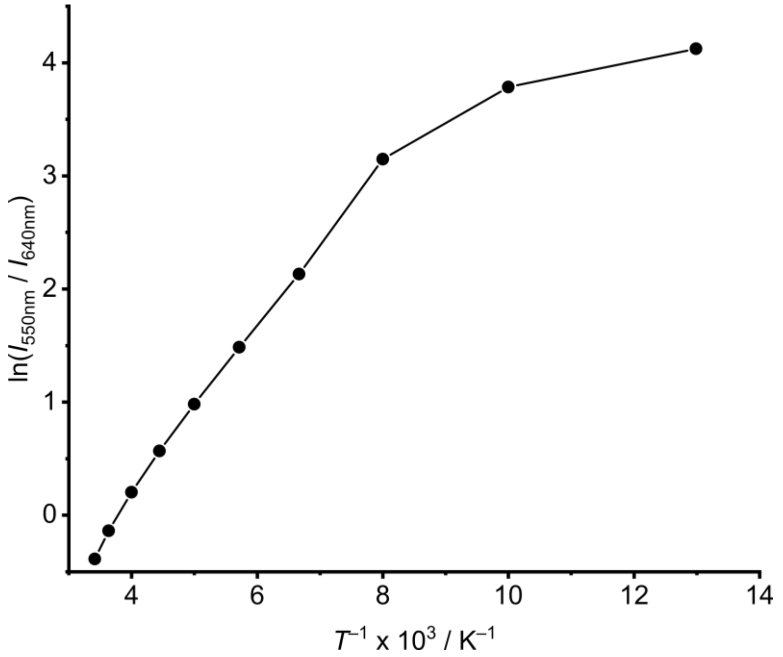

Figure S29. Logarithmic luminescence intensity ratio between the intensities at *l* = 550 nm and *l* = 640 nm plotted against the reciprocal temperature in the solid state.

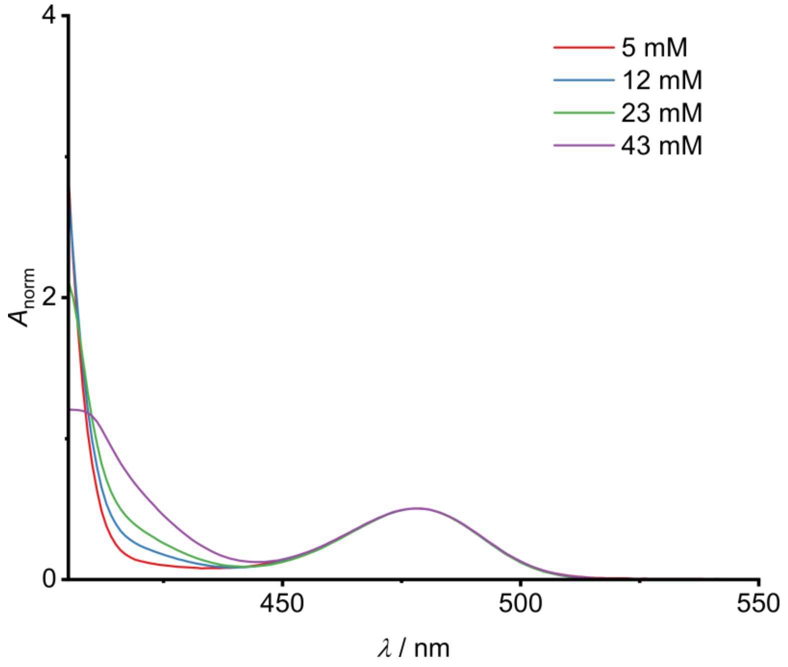

Figure S30. UV/Vis absorption spectra of **Sn** in 3-methylpentane at 77 K, normalised to the $S_0 \rightarrow T_1$ absorption band at various concentrations.

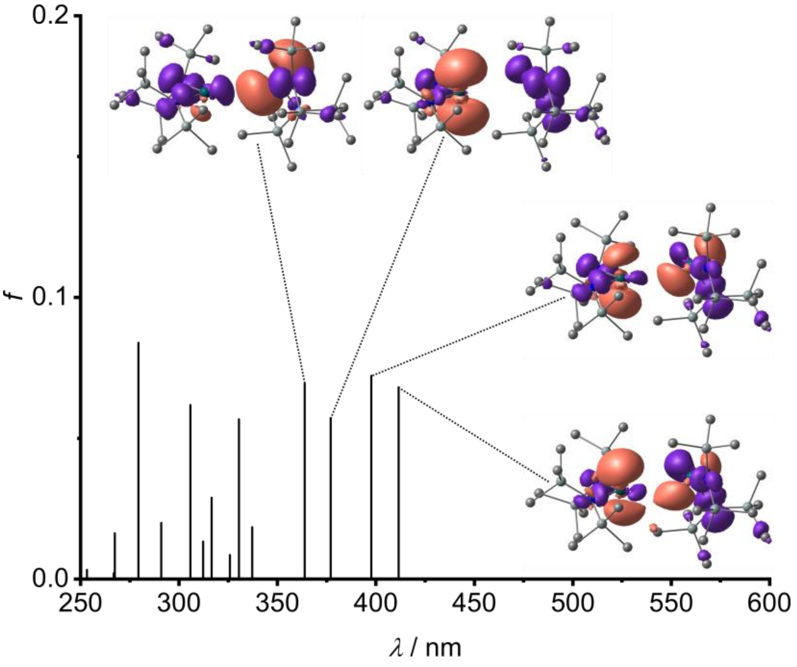

Figure S31. TDDFT calculated spectrum of the geometry optimised dimer ***cis*-^{1}[Sn···Sn]** with difference electron densities of selected transitions (isosurface value 0.002 a.u., purple = electron loss, orange = electron gain. Hydrogen atoms omitted. CPCM(hexane)-RIJCOSX-B3LYP-D3BJ-SARC/J-ZORA/def2-TZVPP/SARC-ZORA-TZVPP(Sn)).

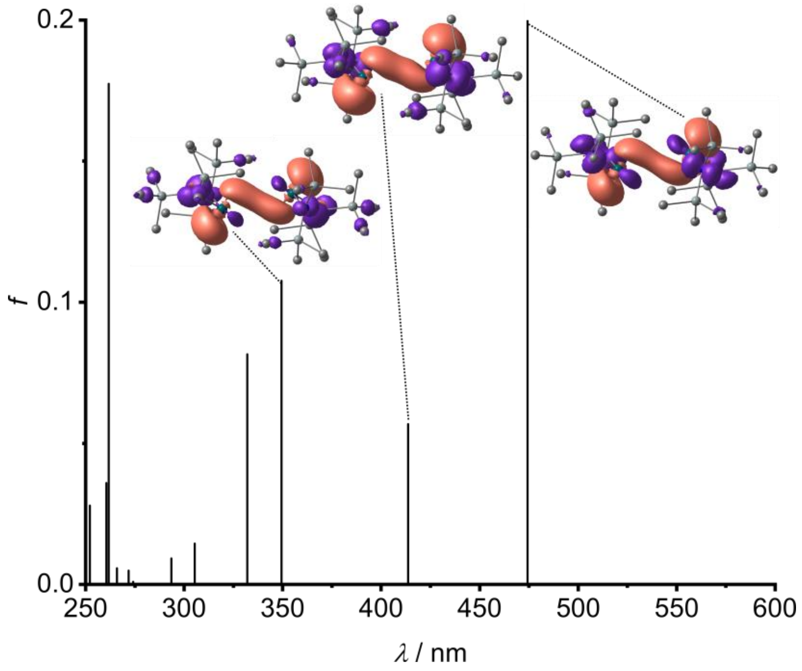

Figure S32. TDDFT calculated spectrum of the geometry optimised dimer ***trans*-[1][Sn···Sn]** with difference electron densities of selected transitions (isosurface value 0.002 a.u., purple = electron loss, orange = electron gain. Hydrogen atoms omitted. CPCM(hexane)-RIJCOSX-B3LYP-D3BJ-SARC/J-ZORA/def2-TZVPP/SARC-ZORA-TZVPP(Sn)).

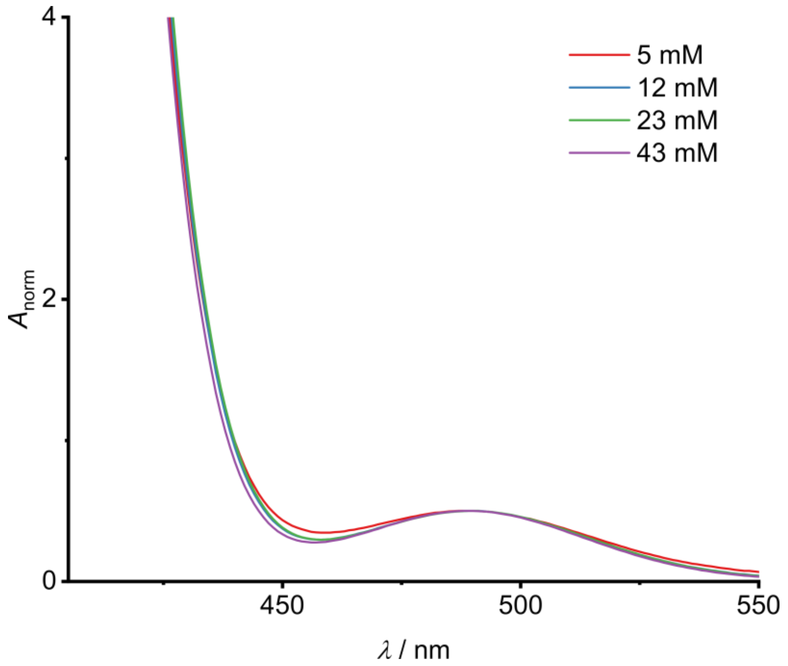

Figure S33. UV/Vis absorption spectra of **Sn** in 3-methylpentane at 293 K, normalised to the $S_0 \rightarrow T_1$ absorption band at various concentrations.

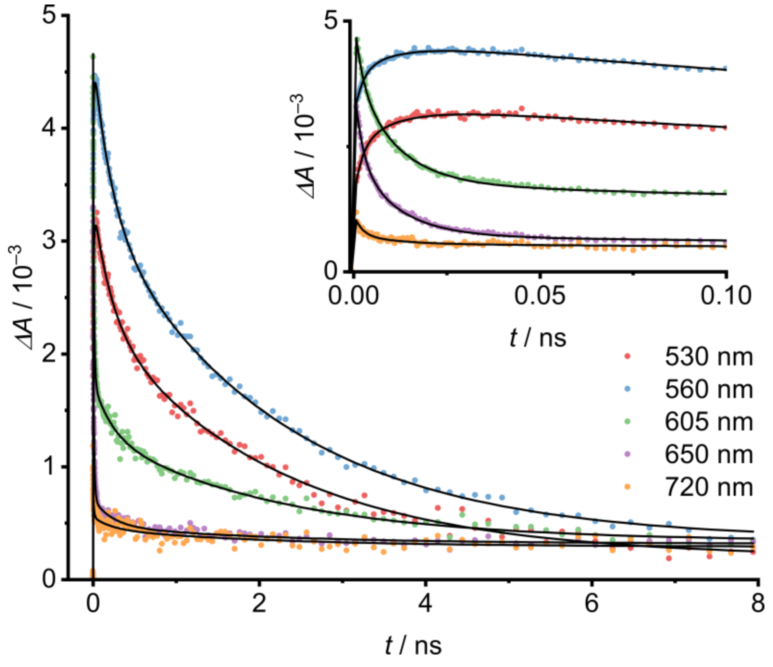

Figure S34. TA decay curves of a sample of **Sn** in 3-methylpentane (c = 22 mM) at 77 K upon λ_{pump} = 430 nm excitation at selected wavelengths with multiexponential fits obtained from global analysis (black curves, τ_1 = 2.2 ps, τ_2 = 11 ps, τ_3 = 222 ps, τ_4 = 2.2 ns, τ_5 = non decaying, sequential model). The inset shows a zoom in the 0−0.1 ns range.

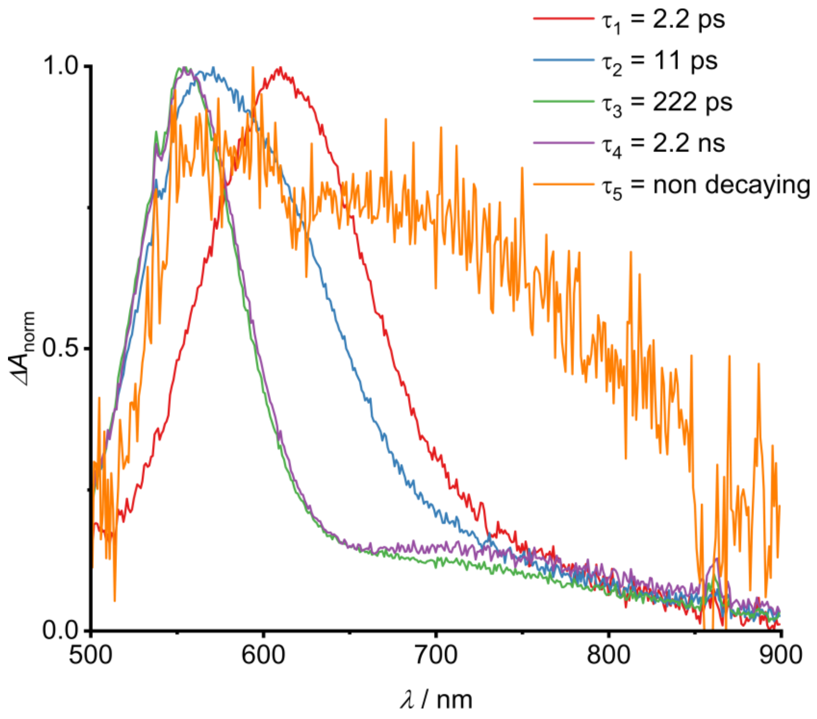

Figure S35. Evolution associated difference absorption spectra of **Sn** (c = 22 mM) in 3-methylpentane at 77 K after λ_{pump} = 430 nm excitation obtained from global analysis with corresponding lifetimes of the components of a sample.

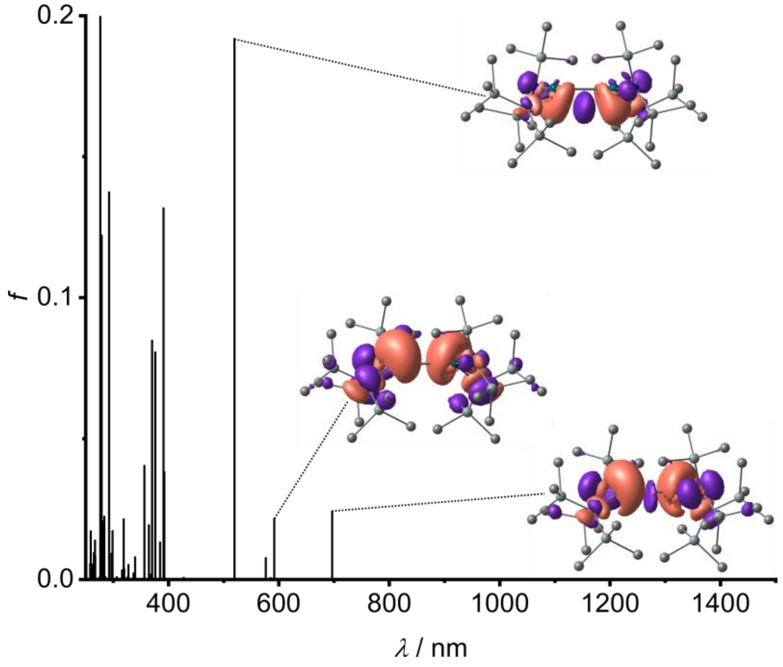

Figure S36. TDDFT calculated spectrum of the geometry optimised triplet excimer ***cis*-^{3}[Sn$_2$]** (isosurface value 0.002 a.u., purple = electron loss, orange = electron gain. Hydrogen atoms omitted. CPCM(hexane)-RIJCOSX-B3LYP-D3BJ-SARC/J-ZORA/def2-TZVPP/SARC-ZORA-TZVPP(Sn)).

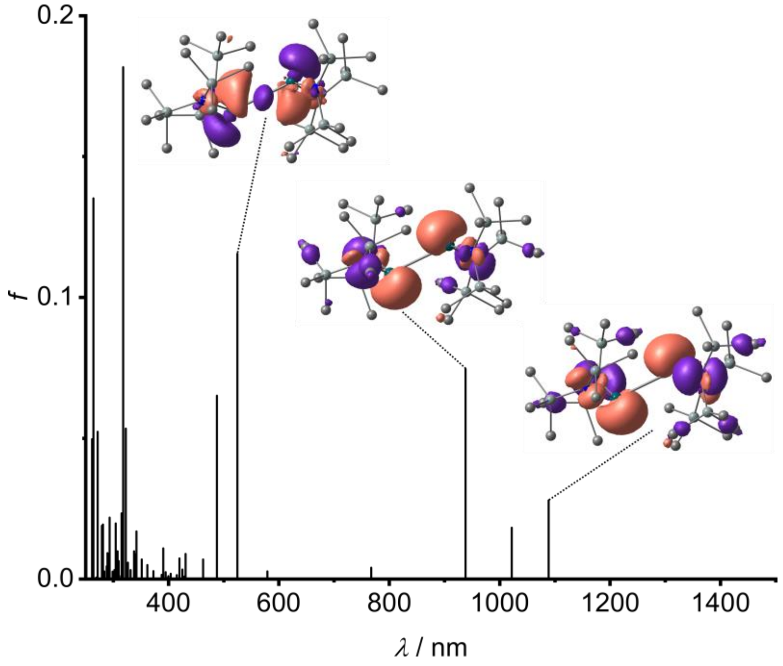

Figure S37. TDDFT calculated spectrum of the geometry optimised triplet excimer ***trans*-^{3}[Sn$_2$]** (isosurface value 0.002 a.u., purple = electron loss, orange = electron gain. Hydrogen atoms omitted. CPCM(hexane)-RIJCOSX-B3LYP-D3BJ-SARC/J-ZORA/def2-TZVPP/SARC-ZORA-TZVPP(Sn)).

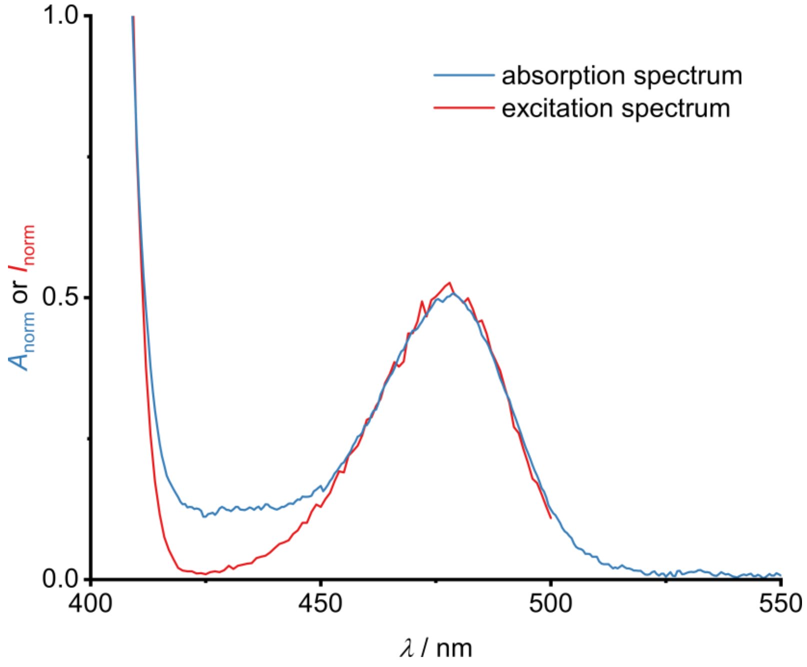

Figure S38. Excitation spectrum of **Sn** in 3-methylpentane at 77 K (λ_{em} = 560 nm, c = 0.42 mM, red) and UV/Vis absorption spectrum of **Sn** in 3-methylpentane at 77 K (c = 0.36 mM, blue).

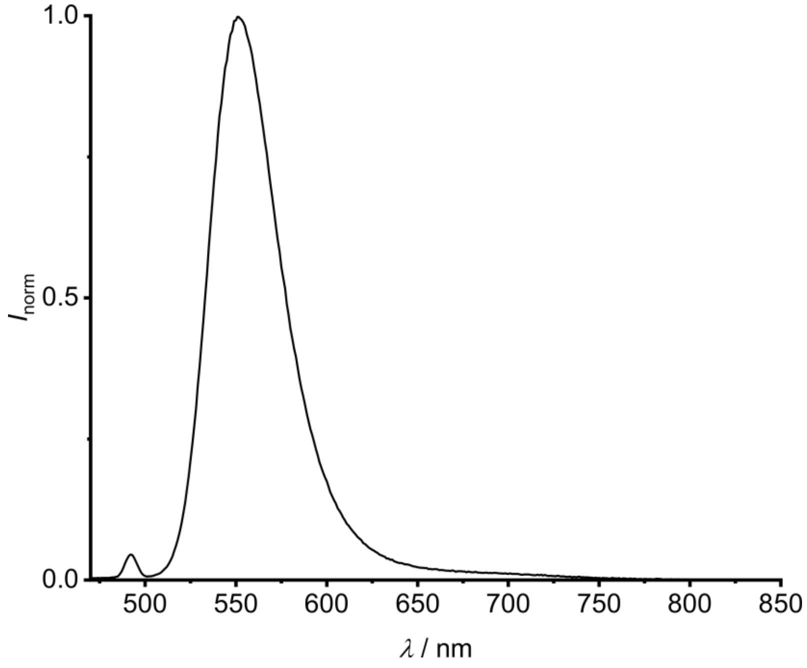

Figure S39. Emission spectrum of **Sn** in 3-methylpentane at 77 K upon λ_{exc} = 430 nm excitation.

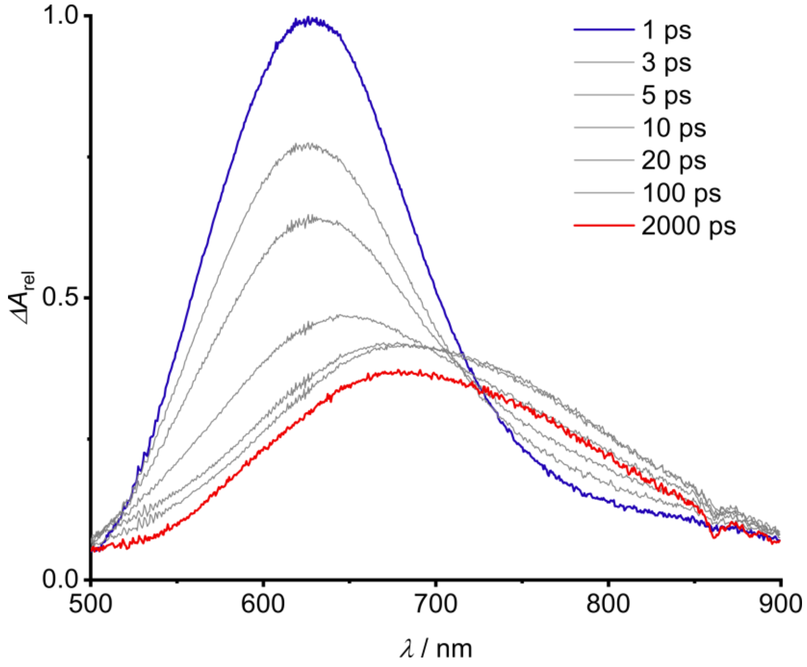

Figure S40. fs-Transient absorption spectra of **Sn** in 3-methylpentane at 293 K upon λ_{pump} = 430 nm excitation at c = 22 mM.

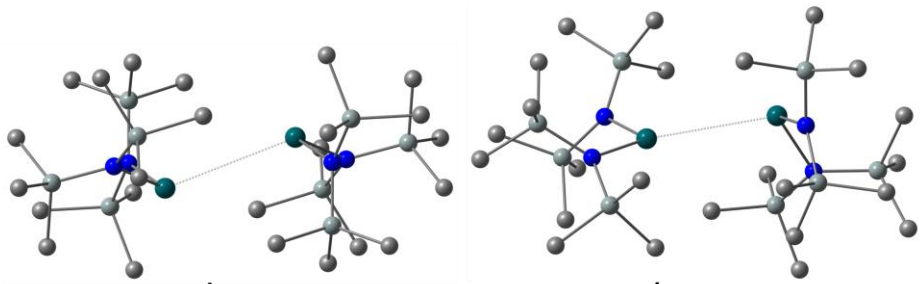

***trans*-¹[Sn···Sn]** ***cis*-¹[Sn···Sn]**

Figure S41. DFT calculated dimers of two **Sn** molecules in the trans-bent ***trans*-¹[Sn···Sn]** and cis-bent ***cis*-¹[Sn···Sn]** orientation. Hydrogen atoms omitted. CPCM(hexane)-RIJCOSX-B3LYP-D3BJ-SARC/J-ZORA/def2-TZVPP/SARC-ZORA-TZVPP(Sn)).

Table S4. Selected molecular orbitals of the model $[SnH_2]_2$ in the triplet state at C_{2v} and C_{2h} symmetry and in the GS at D_{2h} symmetry, displayed at an isosurface value of 0.06 a.u.. Mulliken symbol of the orbital character given in the respective point group. CPCM(hexane)-RIJCOSX-B3LYP-D3BJ-SARC/J-ZORA/def2-TZVPP/SARC-ZORA-TZVPP(Sn)).

C_{2v}	D_{2h}	C_{2h}
b_1	$\sigma^*(b_{3u})$	b_u
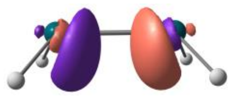	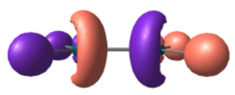	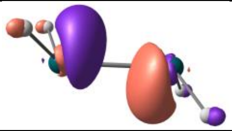
b_1	$\pi^*(b_{1g})$	a_g
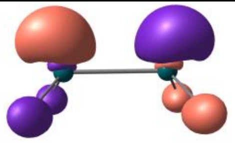	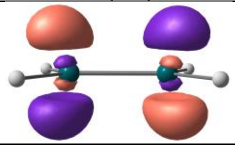	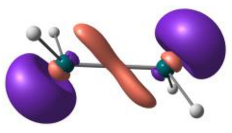
a_1	$\pi(b_{2u})$	b_u

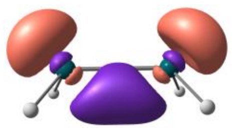	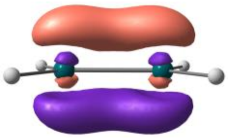	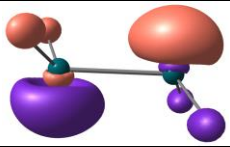
a_1	$\sigma(a_g)$	a_g
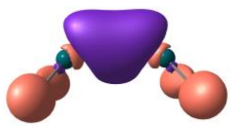	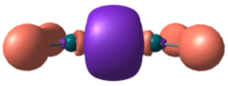	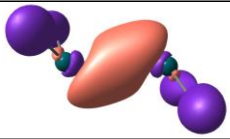

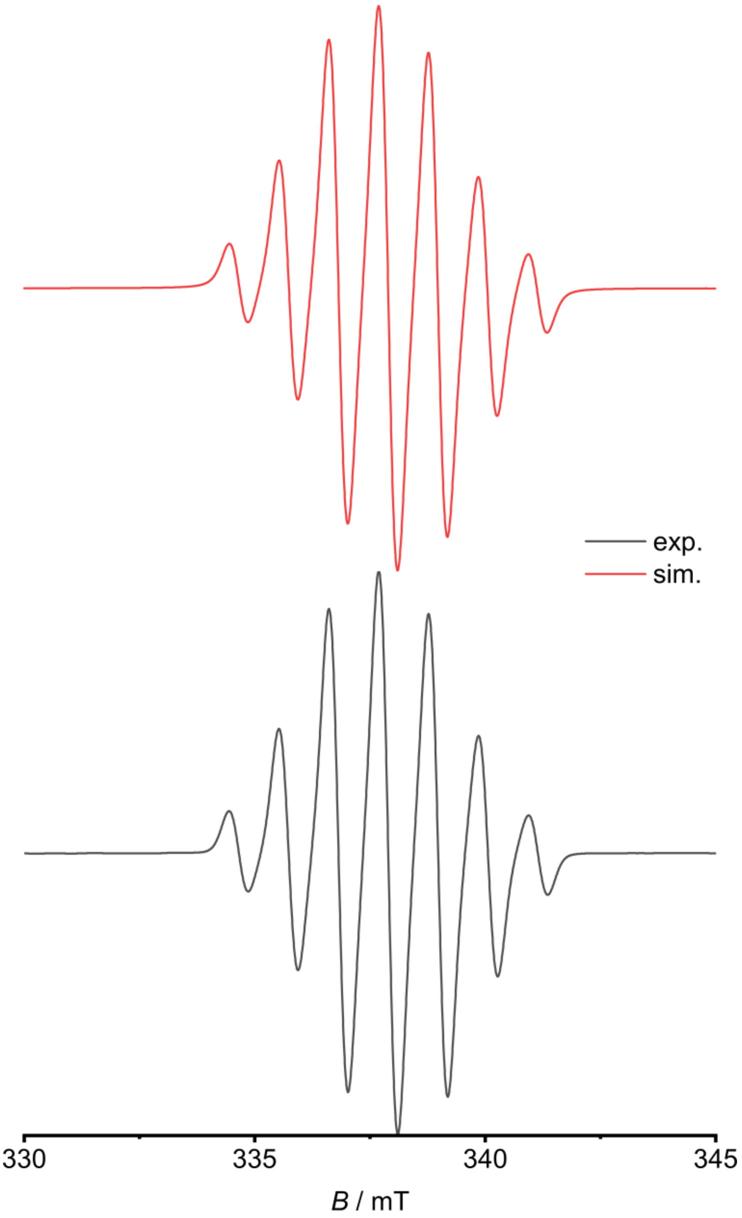

Figure S42. X-band cw-EPR spectrum of •Sn[N(SiMe$_3$)$_2$]$_3$ in *n*-pentane at 293 K (black) and simulated spectrum (red) in the isotropic, fast motional regime with g = 1.996, A(^{14}N) = 30.21 MHz with peak-to-peak Gaussian/Lorentzian line widths 0.38/0.06 mT. The $^{117/119}$Sn satellites could not be detected, being outside the instrument's spectral window.

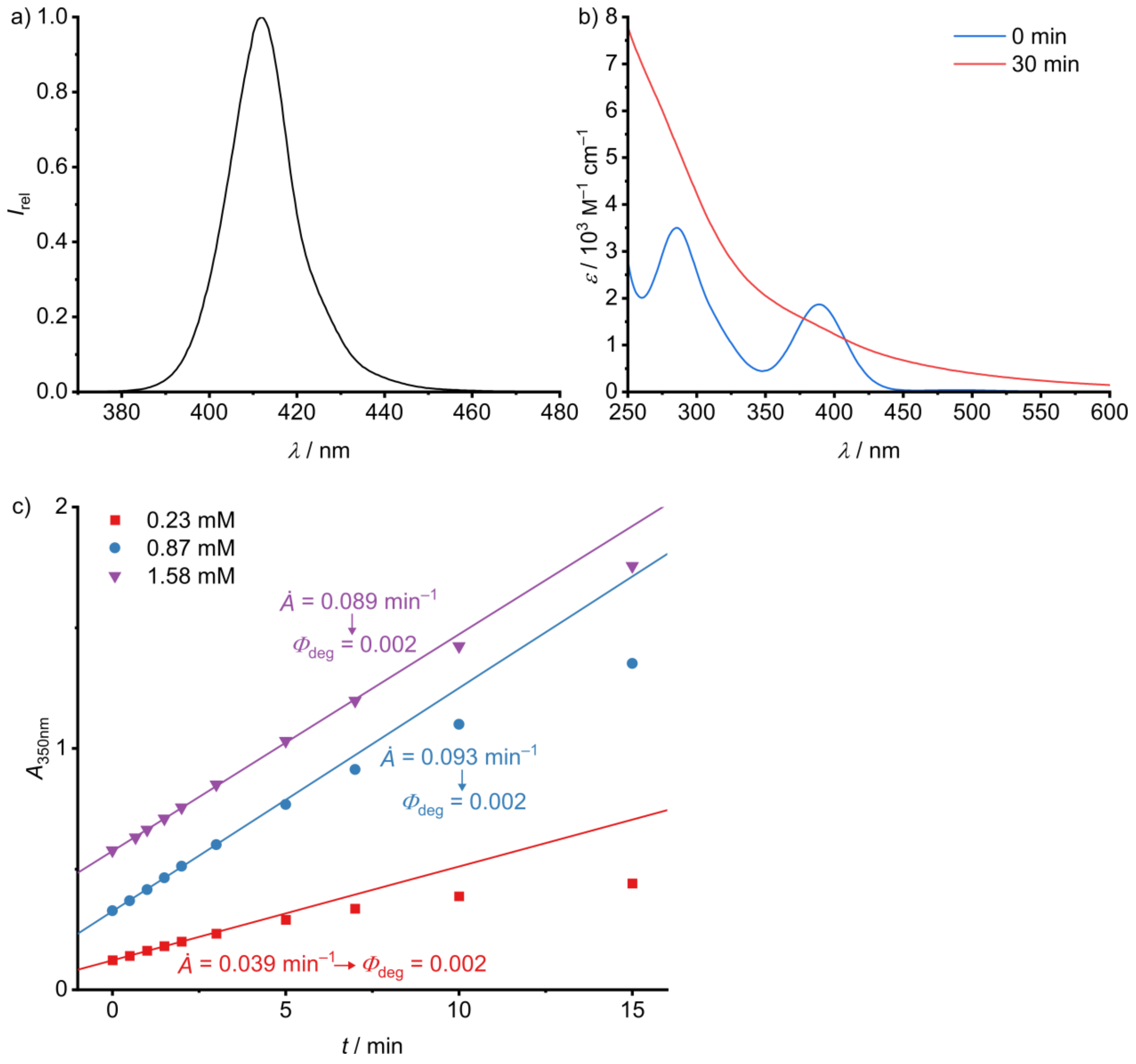

Figure S43. a) Emission spectrum of the used UHP-LED, b) UV/Vis absorption spectrum of **Sn** at t = 0 min in blue and the UV/Vis absorption spectrum of the photolysis product in *n*-pentane after complete conversion t = 30 min in red. c) UV/Vis absorption versus time plots of **Sn** in *n*-pentane at λ = 350 nm of photolysis measurements at c = 0.23, 0.87 and 1.58 mM with linear fits of the data points within the 0−2 min time interval (5 data points).

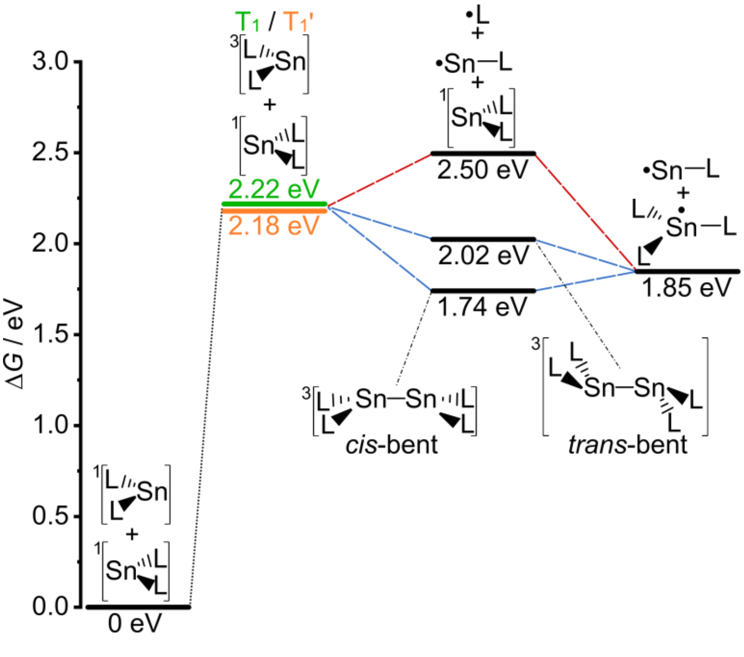

Figure S44. Energy profile diagram with Gibbs free energies in eV for the •Sn[N(SiMe$_3$)$_2$]$_3$ radical formation, starting from two molecules of **Sn** in the GS via the T_1/T_1' states after single molecule excitation with Sn−L bond homolysis and •L radical to **Sn** addition (mechanism A, red path) or via excimer formation with ligand migration and dissociation (mechanism B, blue paths). CPCM(hexane)-RIJCOSX-B3LYP-D3BJ-SARC/J-ZORA/def2-TZVPP/SARC-ZORA-TZVPP(Sn)).

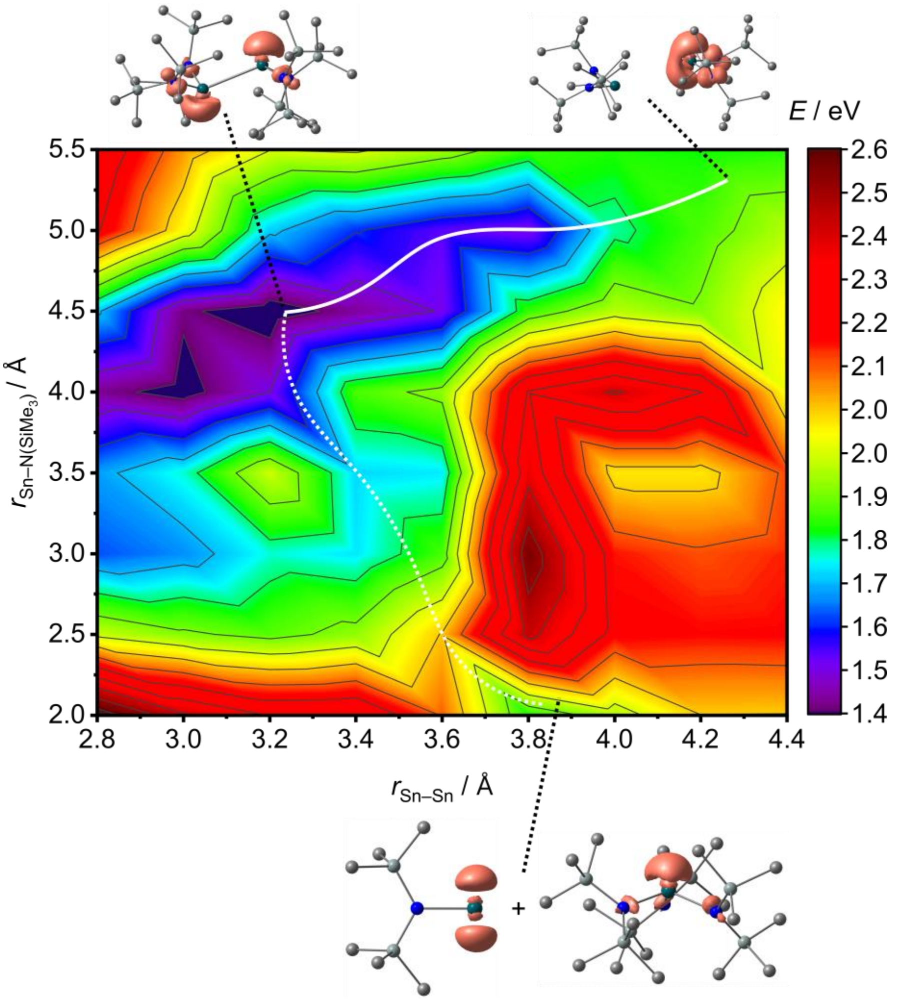

Figure S45. Contour plot of 2D relaxed potential energy surface scans as projection along the Sn−Sn distance and the Sn−N^{HMDS} transfer on the triplet hypersurface of **Sn** with selected geometry optimised molecular structures with spin densities (isosurface value 0.01 a.u.). Energies are given vs. the respective singlet ground state energy (Figure S46). Hydrogen atoms omitted. CPCM(hexane)-RIJCOSX-B3LYP-D3BJ-SARC/J-ZORA/def2-TZVPP/SARC-ZORA-TZVPP(Sn)).

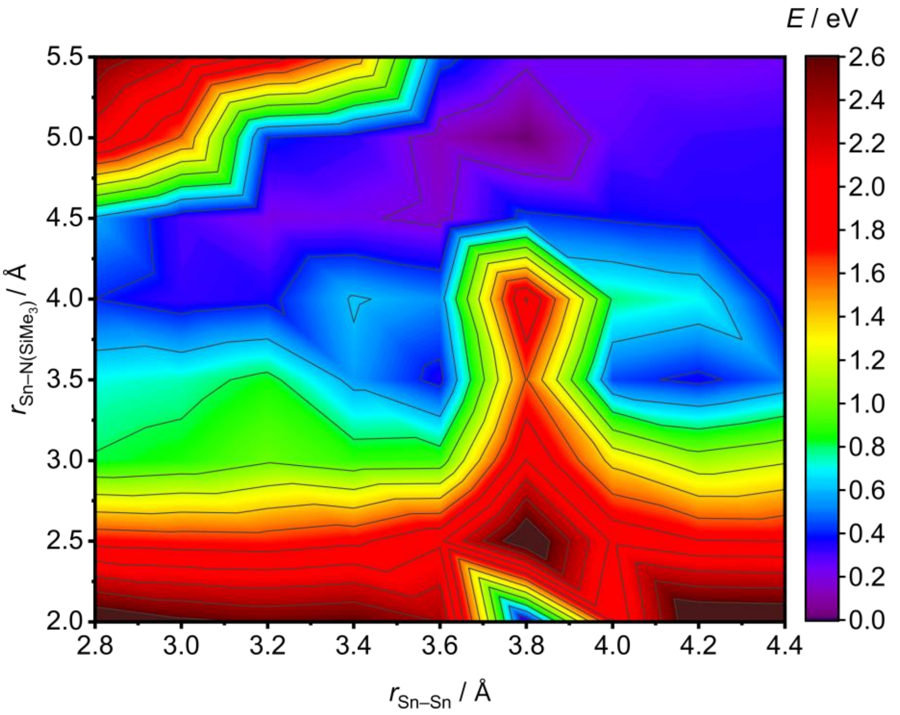

Figure S46. Contour plot of 2D relaxed potential energy surface scans as projection along the Sn−Sn distance and the Sn−N^{HMDS} transfer on the singlet hypersurface of **Sn**. CPCM(hexane)-RIJCOSX-B3LYP-D3BJ-SARC/J-ZORA/def2-TZVPP/SARC-ZORA-TZVPP(Sn)).

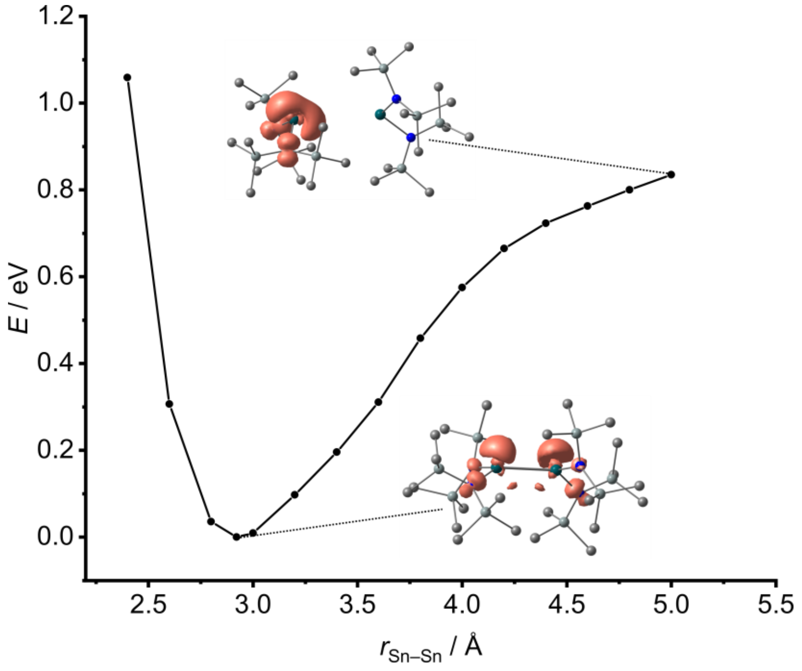

Figure S47. Relaxed potential energy surface scan as projection along the Sn−Sn distance of the ***cis*-^{3}[Sn]** excimer. Hydrogen atoms omitted. Spin densities of selected geometries with isosurface value of 0.01 a.u.. CPCM(hexane)-RIJCOSX-B3LYP-D3BJ-SARC/J-ZORA/def2-TZVPP/SARC-ZORA-TZVPP(Sn)).

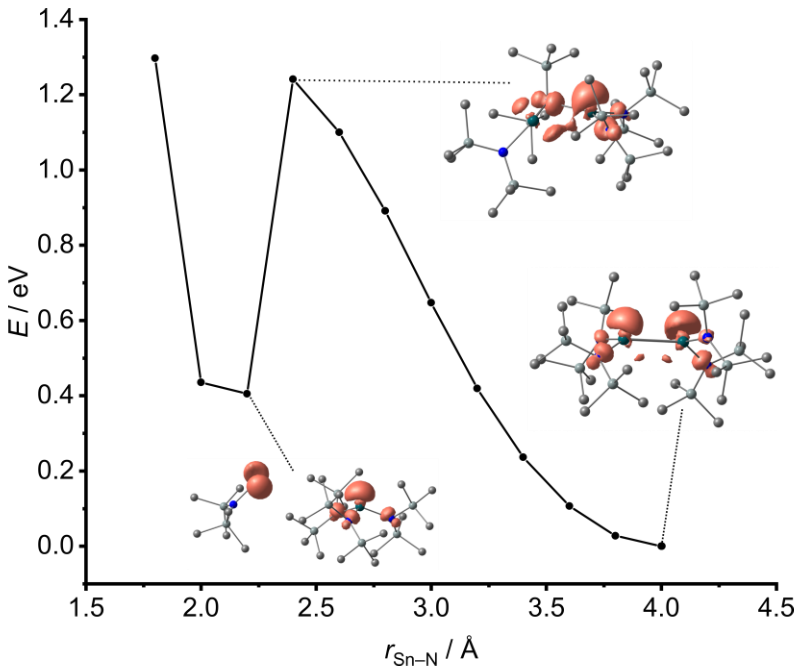

Figure S48. Relaxed potential energy surface scan as projection along the Sn−N[HMDS] transfer of the ***cis*-^{3}[Sn]** excimer. Hydrogen atoms omitted. Spin densities of selected geometries with isosurface value of 0.01 a.u.. CPCM(hexane)-RIJCOSX-B3LYP-D3BJ-SARC/J-ZORA/def2-TZVPP/SARC-ZORA-TZVPP(Sn))

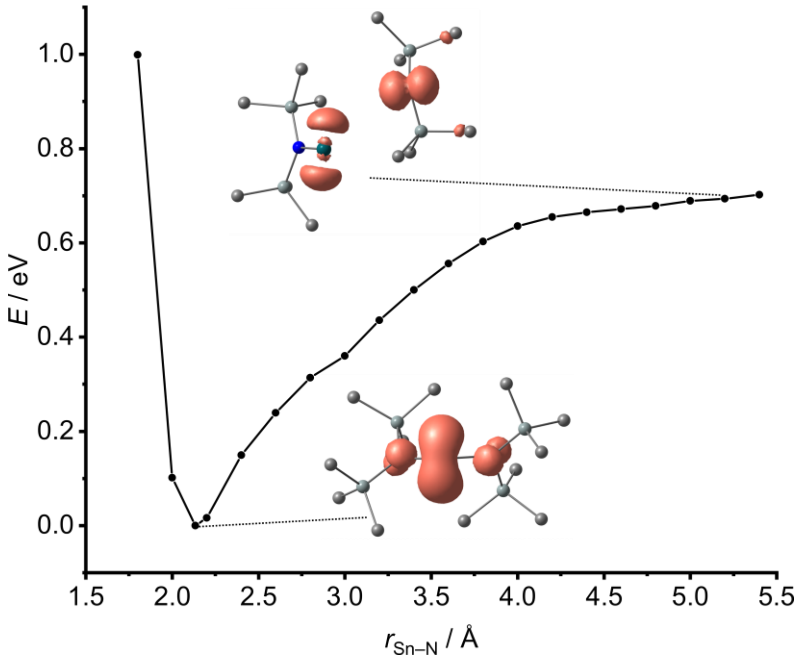

Figure S49. Relaxed potential energy surface scan as projection along the Sn−N[HMDS] distance of **^{3}Sn**. Hydrogen atoms omitted. Spin densities of selected geometries with isosurface value of 0.01 a.u.. CPCM(hexane)-RIJCOSX-B3LYP-D3BJ-SARC/J-ZORA/def2-TZVPP/SARC-ZORA-TZVPP(Sn)).

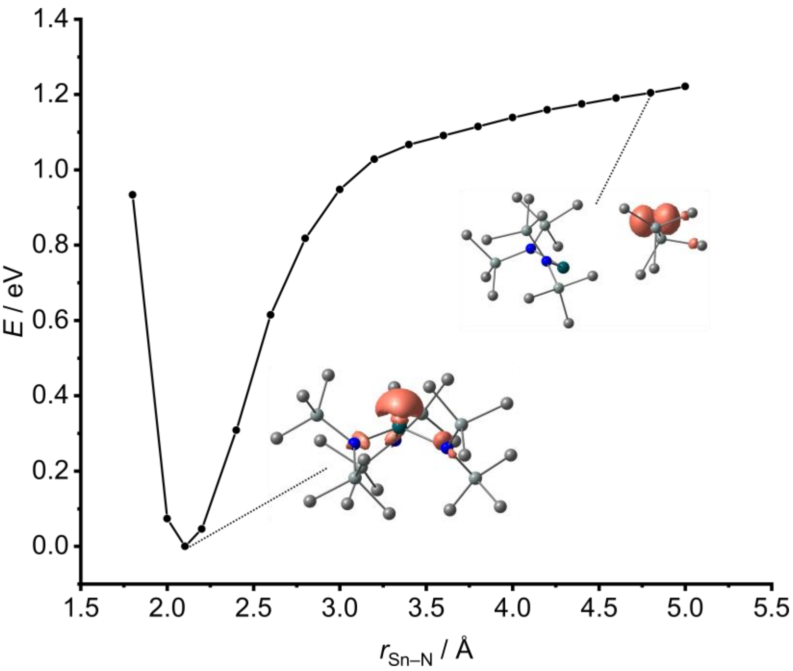

Figure S50. Relaxed potential energy surface scan as projection along the Sn−N^{HMDS} distance of the **•Sn[N(SiMe$_3$)$_2$]$_3$** radical. Hydrogen atoms omitted. Spin densities of selected geometries with isosurface value of 0.01 a.u.. CPCM(hexane)-RIJCOSX-B3LYP-D3BJ-SARC/J-ZORA/def2-TZVPP/SARC-ZORA-TZVPP(Sn)).

[1] G. R. Fulmer, A. J. M. Miller, N. H. Sherden, H. E. Gottlieb, A. Nudelman, B. M. Stoltz, J. E. Bercaw, K. I. Goldberg, *Organometallics* **2010**, *29*, 2176–2179.
[2] C. Müller, T. Pascher, A. Eriksson, P. Chabera, J. Uhlig, *J. Phys. Chem. A* **2022**, *126*, 4087–4099.
[3] S. Stoll, A. Schweiger, *J. Magn. Reson.* **2006**, *178*, 42–55.
[4] F. Neese, *WIREs Comput. Mol. Sci.* **2012**, *2*, 73–78.
[5] F. Neese, *WIREs Comput. Mol. Sci.* **2022**, *12*, e1606.
[6] Lee, Yang, Parr, *Phys. Rev. B Condens.* **1988**, *37*, 785–789.
[7] B. Miehlich, A. Savin, H. Stoll, H. Preuss, *Chem. Phys. Lett.* **1989**, *157*, 200–206.
[8] A. D. Becke, *J. Chem. Phys.* **1993**, *98*, 5648–5652.
[9] F. Weigend, R. Ahlrichs, *Phys. Chem. Chem. Phys.* **2005**, *7*, 3297–3305.
[10] J. D. Rolfes, F. Neese, D. A. Pantazis, *J. Comput. Chem.* **2020**, *41*, 1842–1849.
[11] F. Weigend, *Phys. Chem. Chem. Phys.* **2006**, *8*, 1057–1065.
[12] D. A. Pantazis, X.-Y. Chen, C. R. Landis, F. Neese, *J. Chem. Theory Comput.* **2008**, *4*, 908–919.
[13] D. A. Pantazis, F. Neese, *J. Chem. Theory Comput.* **2009**, *5*, 2229–2238.
[14] D. A. Pantazis, F. Neese, *Theor. Chem. Acc.* **2012**, *131*, 1292.
[15] F. Neese, F. Wennmohs, A. Hansen, U. Becker, *Chem. Phys.* **2009**, *356*, 98–109.
[16] R. Izsák, F. Neese, *J. Chem. Phys.* **2011**, *135*, 144105.
[17] C. van Wüllen, *J. Chem. Phys.* **1998**, *109*, 392–399.
[18] E. van Lenthe, E. J. Baerends, J. G. Snijders, *J. Chem. Phys.* **1993**, *99*, 4597–4610.
[19] S. Miertuš, E. Scrocco, J. Tomasi, *Chem. Phys.* **1981**, *55*, 117–129.
[20] V. Barone, M. Cossi, *J. Phys. Chem. A* **1998**, *102*, 1995–2001.
[21] S. Grimme, J. Antony, S. Ehrlich, H. Krieg, *J. Chem. Phys.* **2010**, *132*, 154104 1-19.
[22] S. Grimme, S. Ehrlich, L. Goerigk, *J. Comput. Chem.* **2011**, *32*, 1456–1465.
[23] F. Neese, *J. Chem. Phys.* **2005**, *122*, 34107.
[24] F. Plasser, *J. Chem. Phys.* **2020**, *152*, 84108.
[25] P. J. Davidson, D. H. Harris, M. F. Lappert, *Dalton Trans.* **1976**, 2268–2274.
[26] M. J. S. Gynane, D. H. Harris, M. F. Lappert, P. P. Power, P. Rivière, M. Rivière-Baudet, *J. Chem. Soc., Dalton Trans.* **1977**, 2004–2009.
[27] C. Glock, S. Krieck, M. Westerhausen, C. M. Lavin, M. M. Gillett-Kunnath, K. Ruhlandt, M. S. Hill, M. D. Anker, A. S. S. Wilson, C. Weetman et al. in *Inorganic Syntheses* (Ed.: P. P. Power), John Wiley & Sons, Inc, Hoboken, NJ, USA, **2018**, pp. 15–31.
[28] A. Schulz, M. Thomas, A. Villinger, *Dalton Trans.* **2018**, *48*, 125–132.
[29] D. H. Harris, M. F. Lappert, *J. Chem. Soc. Chem. Commun.* **1974**, 895–896.
